# Hepatitis B virus genotype, mutations, human leukocyte antigen polymorphisms and their interactions in hepatocellular carcinoma: a multi-centre case-control study

**DOI:** 10.1038/srep16489

**Published:** 2015-11-16

**Authors:** Juan Wen, Ci Song, Deke Jiang, Tianbo Jin, Juncheng Dai, Liguo Zhu, Jiaze An, Yao Liu, Shijie Ma, Na Qin, Cheng Liang, Jiaping Chen, Yue Jiang, Linlin Yang, Jibin Liu, Li Liu, Tingting Geng, Chao Chen, Jie Jiang, Jianguo Chen, Fengcai Zhu, Yefei Zhu, Long Yu, Hongbing Shen, Xiangjun Zhai, Jianfeng Xu, Zhibin Hu

**Affiliations:** 1 Department of Epidemiology and Biostatistics, Jiangsu Key Lab of Cancer Biomarkers, Prevention and Treatment, Jiangsu Collaborative Innovation Center For Cancer Personalized Medicine, Cancer Center, School of Public Health, Nanjing Medical University, Nanjing, China; 2 Nanjing Maternity and Child Health Care Institute, Nanjing Maternity and Child Health Care Hospital Affiliated with Nanjing Medical University, Nanjing, China; 3 State Key Laboratory of Genetic Engineering, Collaborative Innovation Center for Genetics and Development, School of Life Sciences, Fudan University, Shanghai, China; 4 Ministry of Education Key Laboratory of Contemporary Anthropology, School of Life Sciences, Fudan University, Shanghai, China; 5 Fudan Center for genetic Epidemiology and Center for Genetic Translational Medicine and Prevention, Fudan University, Shanghai, China; 6 School of Life Sciences, Northwest University, Xi’an, China; 7 National Engineering Research Center for Miniaturized Detection Systems, Xi’an, China; 8 Jiangsu Province Center for Disease Prevention and Control, Nanjing, China; 9 Department of Hepatobiliary Surgery, Xijing Hospital, Fourth Military Medical University, Xi’an, China; 10 Pathology Center and Department of Pathology, Soochow University, Suzhou, China; 11 Department of Gastroenterology, Huai’an First People’s Hospital of Nanjing Medical University, Huai’an, China; 12 Department of Hepatobiliary Surgery, Nantong Tumor Hospital, Nantong, China; 13 Digestive Endoscopy Center, the First Affiliated Hospital of Nanjing Medical University, Nanjing, China; 14 Department of Epidemiology, Qidong Liver Cancer Institute, Qidong, China; 15 Tumor Institute, Nantong Tumor Hospital, Nantong, China; 16 Center for Cancer Genomics, Wake Forest University School of Medicine, Winston-Salem, North Carolina, USA; 17 Jiangsu Key Laboratory of Molecular and Translational Cancer Research, Nanjing Medical University Affiliated Cancer Hospital, Cancer Institute of Jiangsu Province, Nanjing, China

## Abstract

Three genome-wide association studies (GWAS) have been conducted on the genetic susceptibility of hepatitis B virus (HBV)-related hepatocellular carcinoma (HCC), two of which consistently identified tagging single nucleotide polymorphisms (SNPs) around *HLA-DQ/DR*. In contrast, large multi-centre association studies between HBV genotype, mutations and the risk of HCC are relatively rare, and their interactions with host variants are even less. We performed a multi-centre study of 1,507 HBV-related HCC cases and 1,560 HBV persistent carriers as controls to evaluate the effects of HBV genotype, mutations, GWAS-identified *HLA-DQ/DR* SNPs (rs9272105 and rs9275319) and their interactions on HCC risk. We found HBV genotype C was more frequent in HBV-related HCC. And 11 HBV hotspot mutations were independently and significantly associated with HCC risk. We also detected significant interactions of rs9272105 with both the HBV genotype and mutations. Through stepwise regression analysis, HBV genotype, the 11 mutations, *HLA-DQ/DR* SNPs, and the interaction of rs9272105 with mutation A1752G were all entered into the HCC prediction model, and the area under the curve for the panel including the *HLA-DQ/DR* SNPs, HBV genotype and mutations was 0.840. The HBV genotype, the mutations and the *HLA-DQ/DR* SNPs may serve as biomarkers for the surveillance of HBV persistent carriers.

Hepatocellular carcinoma (HCC) is the second leading cause of cancer-related deaths worldwide, with the incidence on the rise both in developed and developing countries^1^,^2^. HCC development is influenced by complex factors including viral infection, environmental factors^3^, and genetic makeup, with most studies having identified susceptibility loci at the human leukocyte antigen (HLA) class II region at 6p21^4^,^5^,^6^.

Currently, three genome-wide association studies (GWAS), all from China, have been conducted on hepatitis B virus (HBV)-related HCC, two of which consistently identified *HLA-DQ/DR* as susceptibility loci^5^,^6^. Of the two independent GWAS, one study with 1,538 cases and 1,465 controls for GWA scan identified the single nucleotide polymorphism (SNP) rs9272105 (located between *HLA-DQA1* and *HLA-DRB1*)^5^, while the other study with 1,161 HCC cases and 1,353 controls for GWA scan identified the SNP rs9275319 at *HLA-DQ*^6^. These findings highlight the importance of HLA-DQ/DR molecules in the development of HBV-related HCC.

In contrast, large multi-centre studies on the association between HBV genotype, mutations and HCC risk are relatively rare, especially regarding their interactions with host genetic variants. HBV has been classified into different genotypes according to a sequence divergence of >8% in the entire genome, and the genotypes are further separated into subgenotypes if the divergence in the nucleotide sequence is between 4 and 8%^7^,^8^. The HBV genotypes and subgenotypes have distinct geographic distributions and have been implicated to differ with regard to clinical liver diseases, disease outcomes, and responses to interferon treatment^9^,^10^,^11^,^12^. In East Asia, although HBV genotypes B and C are endemic, the HBV genotype frequency varies in these areas^13^. Because of the relatively small study sample sizes, the low success rates of HBV typing and the different study designs, the effect of HBV genotype on the outcomes of HBV persistent infection also varied greatly^14^,^15^,^16^,^17^,^18^,^19^. The basal core promoter, which is regulated by the enhancer II to a great extent, controls the transcription of precore mRNA^20^. The precore protein is processed to produce the secreted hepatitis B e antigen (HBeAg), which indicates active viral replication and is associated with an increased risk of HCC^21^,^22^,^23^. Three small-scale longitudinal studies with less than 50 cases demonstrated that some of the mutations in the enhancer II/basal corepromoter/precore (EnhII/BCP/PC) region would occur years before a diagnosis of HCC is made and gradually accumulate during the development of HCC^24^,^25^,^26^.

HBV mutations are most likely selected via virus-immune interactions in the inflammatory microenvironment. Therefore, we performed a large multi-centre study to evaluate the effects of HBV genotype, mutations in the EnhII/BCP/PC region, GWAS-identified *HLA-DQ/DR* SNPs (rs9272105 and rs9275319) and their interactions on HCC risk. Furthermore, we evaluated the risk prediction effects of these factors in HBV-related HCC.

## Results

### Baseline characteristics of the participants

Selected characteristics of the 1,507 HBV-related HCC patients and the 1,560 HBV persistent carriers are described in Supplementary Table S2. As expected, there were similar distributions of age and gender between the HCC patients and the HBV persistent carriers (*P* = 0.835 and 0.687, respectively). In addition, similar distributions of age and gender were also seen between the cases and controls of the additional sample set from Xi’an (Supplementary Table S3).

### Distribution of HBV genotypes and subgenotypes

Among the participants of this study, the HBV genotypes B, C, BC (coinfection) and D and the subgenotypes B2, C1, C2, B2C1, B2C2, B2C1C2 and C1C2 were identified through nested multiplex PCR and sequencing. All the amplicons were of the size expected for each genotype and subgenotype, as shown in Supplementary Fig. S1. In case where the genotype was identified but the subgenotype was not, the HBV subgenotype was identified by sequencing and aligning, especially for C3 and C4. The sequencing peak chart of all the genotypes and subgenotypes is shown in Supplementary Fig. S2.

As shown in Table 1, we found 629 (41.0%) subjects infected with HBV genotype B, 375 subjects (24.4%) with genotype BC, 529 subjects (34.4%) with genotype C, and 3 subjects (0.2%) with genotype D among the HBV persistent carriers, whereas there were 159 subjects (10.8%) with HBV genotype B, 121 subjects (8.2%) with genotype BC, 1,186 subjects (80.6%) with genotype C, and 5 subjects (0.3%) with genotype D among the HCC patients. Although the frequencies of the HBV genotypes B and BC (coinfection) were varied among the studied areas (Supplementary Table S4), the frequency of genotype C was consistently higher in HBV-related HCC subjects (80.6% among HCC vs. 34.4% among HBV persistent carriers, Table 1). Compared with subjects infected with HBV B-related genotypes (B and BC), the subjects infected with non-B genotypes (C and D) had an 8.18-fold increased HCC risk (95% CI = 6.91–9.68). Among the HBV subgenotypes, B2, C2 and B2C2 were predominant. The proportions of subgenotype C2 in HCC patients and HBV persistent carriers were 67.0% and 29.3%, respectively. Similarly, the genotype and subgenotype frequencies also suggested that HBV genotype C and subgenotype C2 were the risk factors for HCC in the Xi’an sample set (Supplementary Table S5).

### Associations of HBV mutations and HBV-related HCC

We successfully amplified and sequenced the EnhII/BCP/PC region from 1,313 (89.3%) HCC patients and 1,413 (92.0%) HBV persistent carriers with HBV genotype results. The wild type nucleotide and hotspot mutations in the EnhII/BCP/PC region are listed in Supplementary Table S6. The sequences of the hotspot mutations are shown in Supplementary Fig.S3. Of those 19 hotspot mutations, C1653T, T1674C/G, A1703G, G1719T, T1727A/G, T1753C, A1762T, G1764A, G1799C, G1899A, G1915A/C and C1969T were significantly associated with an increased risk of HCC, whereas C1673T, A1726C, C1730G and A1752G were significantly associated with a reduced risk of HCC, when adjusted for age and gender (Supplementary Table S7). We then used a conditional logistic regression analysis to test the independence of these hotspot mutations and found that the effects of C1653T, C1673T, T1674C/G, C1730G, A1752G, T1753C, A1762T, G1764A, G1899A, G1915A/C and C1969T on HCC development remained in existence after being conditioned on the other mutations (Supplementary Table S7). After Bonferroni correction, HBV mutations C1653T, T1674C/G, A1752G, T1753C, A1762T, G1764A, G1899A, and C1969T were still significant associated with HCC risk.

Supplementary Table S8 shows the correlations between the hotspot mutations in the EnhII/BCP/PC region of HBV. The results indicated that the HBV mutation C1730G was correlated with T1727A/G and G1799C (r^2^ > 0.800).

### Associations of the *HLA* SNPs with HBV-related HCC and their interactions with HBV genotype and mutations in HCC

The genotype distributions of the SNPs rs9272105 and rs9275319 in the HCC patients and the HBV persistent carriers are described in Supplementary Table S9. The observed genotype frequencies for the two SNPs in the HBV persistent carriers were all in Hardy-Weinberg equilibrium (*P* = 0.641 for rs9272105 and *P* = 0.274 for rs9275319). The logistic regression analyses in the additive genetic model showed that a variant allele of rs9272105 increased the host HCC risk compared to the HBV persistent carriers (adjusted OR = 1.31, 95% CI = 1.18–1.45), whereas a variant allele of rs9275319 was associated with a decreased HCC risk (adjusted OR = 0.66, 95% CI = 0.56–0.78). In addition, the allele frequencies of the two SNPs were similar between the case subjects and the controls from different areas.

**Figure 1:**
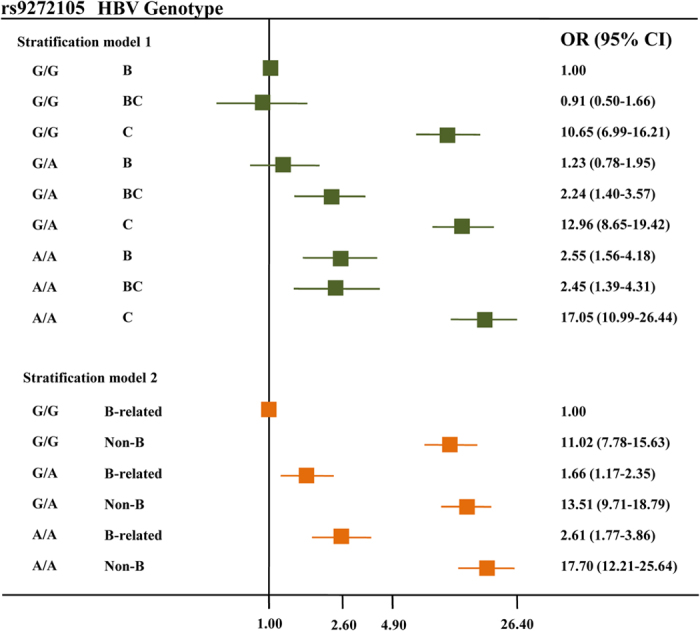
Crossover analysis of the SNP rs9272105-HBV genotype interaction on HCC susceptibility, including stratification model 1 (B, BC and C) and model 2 (B-related and non-B). The B-related genotypes included B and BC, whereas the non-B genotypes included C and D.

Then, subgroup analyses stratified on the HBV genotype model 1 (B, BC and C) and model 2 (B-related and non-B) were conducted on the associations of the *HLA* SNPs with HCC risk (Supplementary Table S10). Similar association strengths were shown between all the subgroups for rs9275319 (*P* > 0.05 for heterogeneity test). Interestingly, a stronger effect of rs9272105 was observed among the subjects infected with B-related genotypes (adjusted OR = 1.61, 95% CI = 1.33–1.95) compared to those infected with non-B genotypes (adjusted OR = 1.26, 95% CI = 1.09–1.46) (*P* = 0.046 for heterogeneity test). A further interaction analysis detected a significant multiplicative interaction between rs9272105 and HBV genotype on HCC risk (*P* = 0.014 for model 1 and *P* = 0.046 for model 2). The crossover analysis suggested that subjects infected with HBV genotype C and carrying the rs9272105 AA genotype had a 17.05-fold increased HCC risk (95% CI = 10.99–26.44) compared with those infected with HBV genotype B and carrying the rs9272105 GG genotype. Additionally, the subjects infected with HBV non-B genotypes and carrying the rs9272105 AA genotype had a more prominent risk effect (adjusted OR = 17.70, 95% CI = 12.21–25.64) compared to those infected with HBV B-related genotypes and carrying the rs9272105 GG genotype (Fig. 1).

**Figure 2:**
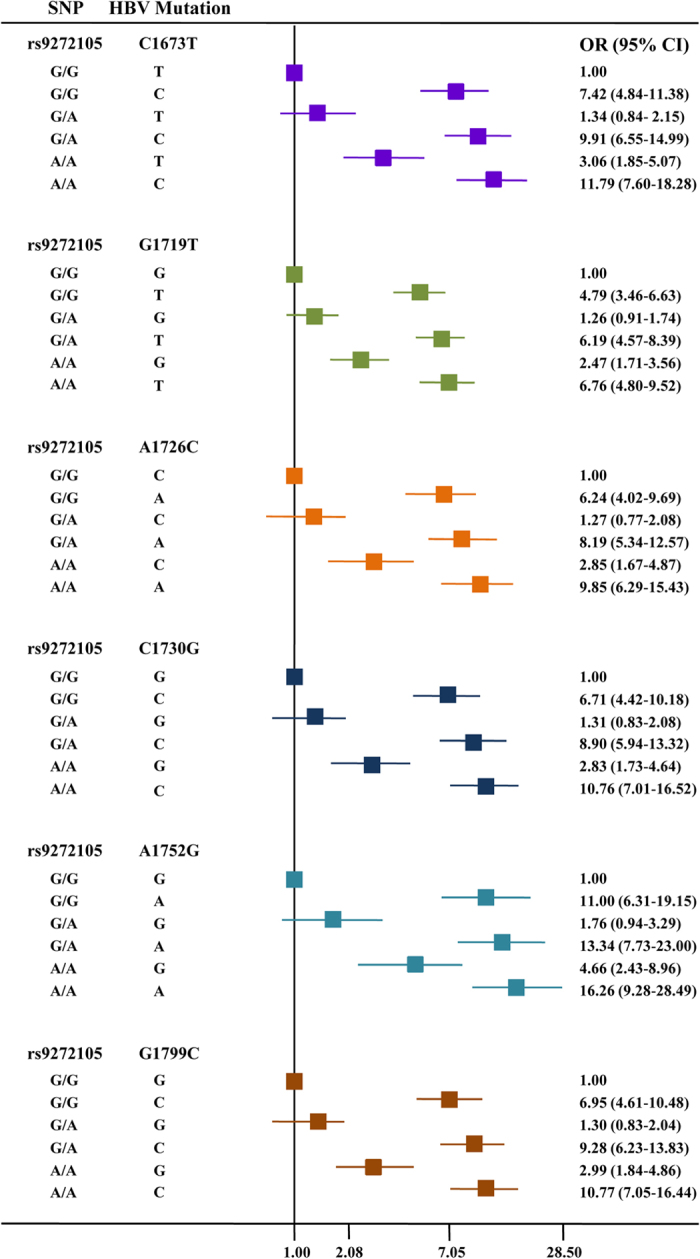
Crossover analysis of the SNP rs9272105-HBV mutations interactions on HCC susceptibility. From top to bottom, the HBV mutations are C1673T, G1719T, A1726C, C1730G, A1752G and G1799C, respectively.

Multiplicative interactions of rs9272105 and rs9275319 with all the HBV hotspot mutations were also evaluated (Supplementary Table S11). We detected significant interactions between rs9272105 and the HBV mutations C1673T, G1719T, A1726C, C1730G, A1752G and G1799C on HCC risk (*P* < 0.05). As shown in Fig. 2, rs9272105 multiplicatively interacted with C1673T, G1719T, A1726C, C1730G, A1752G and G1799C. Thus, the effects of the HBV mutations on HCC risk depended on the HLA genetic background. However, we did not observe any significant interaction between rs9275319 and HBV genotype or mutations on HCC risk.

### Performance of the panels of the *HLA* SNPs, HBV genotype and mutations in the prediction of HCC risk

**Figure 3:**
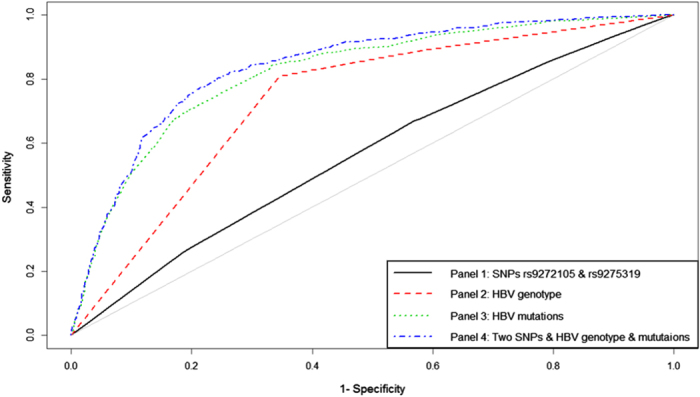
The discriminative ability of four panels between the HBV-related HCC patients and the HBV persistent carriers was evaluated by a ROC curve analysis. Black line, panel of the SNPs rs9272105 and rs9275319 (AUC = 0.565, sensitivity = 67.0%, specificity = 43.1%); red line, HBV genotype panel (AUC = 0.736, sensitivity = 80.9%, specificity = 65.5%); green line, HBV mutations panel (AUC = 0.821, sensitivity = 70.9%, specificity = 80.1%); blue line, the panel including the two SNPs, HBV genotype and mutations (AUC = 0.840, sensitivity = 81.3%, specificity = 74.8%).

After a stepwise regression analysis, the HBV genotype, the 11 independent HCC-related mutations, the *HLA* SNPs (rs9272105 and rs9275319) and the interaction of rs9272105 with A1752G were entered into the HCC prediction model (Table 2). Then, we constructed the ROC curve to evaluate the risk prediction performance of the *HLA* SNPs, HBV genotype and mutations for the HCC patients (Fig. 3). The AUC of the panels of the two SNPs, HBV genotype and mutations were 0.565, 0.736 and 0.821, respectively. In fact, the AUC of the panel that combined the two SNPs, HBV genotypes and the 11 mutations was even better than the above panels at 0.840 (sensitivity = 81.3%, specificity = 74.8%). The difference between the AUC of the panel 3 (HBV mutations) and panel 4 (two SNPs, HBV genotypes and mutations) was statistically significant (Z = −5.08, *P* = 3.82 × 10^−7^). For the panel including two SNPs, HBV genotype and mutations, the Hosmer-Lemeshow χ^2^ (8 degrees of freedom) was 11.64 (*P* = 0.168), giving no cause for concern over model fit or calibration.

## Discussion

In this large multi-centre study, we found that HBV genotype C and subgenotype C2 were the risk factors for HCC, and 11 HBV mutations were found to be significantly associated with HCC risk. We also detected significant interactions of *HLA-DQ/DR* rs9272105 with both HBV genotype and mutations, which may imply a potential biological significance forrs9272105. Excitingly, the panel that combined the *HLA-DQ/DR* SNPs, HBV genotypes and mutations provided a high sensitivity and specificity to discriminate the HCC patients from the controls. The HBV carriers who infected with HBV genotype C and carrying the rs9272105 AA genotype, rs9275319 AA genotype and risky nucleotide of the 11 HBV mutations had a relatively high HCC risk, which was useful in screening of high-risk groups of HCC and needed to be validated in further prospective studies.

The development of HCC is a multistage process, and most HCCs arise from chronic hepatitis induced by HBV infection, particularly in China^27^. With the progression of chronic infection, HBV mutations gradually occur^28^,^29^. HBV reverse transcriptase lacks proofreading activity, resulting in an estimated mutation rate of 4.57 × 10^−5^ nucleotide (nt) substitutions per site per year^30^. Inflammatory factors could also promote HBV mutations, and the insufficient immune responses elicited by the HBV antigens select the disease-related HBV mutations during the long-term evolutionary process^31^,^32^. Only the HBV strains/variants best adapted to the host immune system will survive and thrive in liver^33^. The *HLA* system is the name of the locus of genes that encodes the major histocompatibility complex (MHC) in humans. This super-locus contains a large number of genes related to immune system function in humans. HLA class II molecules include three isotypes: HLA-DR, HLA-DQ, and HLA-DP, which have been reported on extensively for their association with HBV infection and hepatocarcinogenesis^5^,^6^,^34^,^35^,^36^. In this study, we detected significant interactions of *HLA-DQA1/DRB1* rs9272105with HBV genotype and mutations on HCC risk. Thus, *HLA- DQ/DR* genetic polymorphisms might affect the outcomes of chronic HBV infection via regulating the immune selection of HBV mutations, thereby affecting the risk of HCC caused by the HBV mutations.

In this study, the HBV mutations A1846T and G1896A, which have been reported to be associated with an increased risk of HCC by several studies^33^,^37^,^38^, were not significantly associated with HCC. This may be due to different study areas, the sample sizes and the adjustment for other HBV mutations. There were also novel HBV mutations, including A1752G, G1915A/C and C1969T, which were found to be associated with HBV-related HCC. In addition, the effect of C1730G was reversed from a protective effect (adjusted OR = 0.18, 95% CI = 0.15–0.22) to a risk effect with borderline significance (adjusted OR = 2.07, 95% CI = 1.02–4.20) after being conditionally adjusted by the other mutations. Thus, the functional effects of these HBV mutations on HCC risk deserve further investigation.

We successfully validated the associations between *HLA* SNPs (rs9272105 and rs9275319) and HCC risk. SNP rs9272105 is located between *HLA-DQA1* and *HLA-DRB1*, and rs9275319 is located between *HLA-DQB1* and *HLA-DQA2*. HLA-DQ and -DR proteins make up the HLA class II complex, an α-β heterodimeric membrane glycoprotein that is expressed on the surface of antigen-presenting cells, such as B lymphocytes, macrophages and den-dritic cells. HLA class II glycoproteins present viral peptides to CD4^+^ T cells and influence the immune responses. Therefore, SNPs in *HLA-DQ* and *-DR* genes may have important roles in immune-mediated diseases, including liver diseases and HCC.

Recently, Cao *et al.* conducted a case-control study (1,108 HCC patients and 1,628 HBV-positive subjects without HCC) to evaluate the effects of *HLA-DP* polymorphisms (four SNPs reported by a GWAS of HBV persistent infection), HBV EnhII/BCP/PC region mutations and their interactions on HCC risk in subjects infected with HBV genotype B or genotype C. They sequenced the HBV EnhII/BCP/PC region successfully from 1,429 (52.2%) of the HBV-infected subjects and found that the interactions of rs9277535 AA with the T1674C/G or G1719T mutation in genotype C significantly decreased HCC risk^33^. The same group also evaluated the effect of the *STAT3* SNPs and their interactions with HBV mutations on HCC risk with a sample size of 1,021 HCC patients and 990 HBV-positive subjects without HCC. In this study, they genotyped three SNPs of *STAT3*and sequenced the HBV EnhII/BCP/PC region successfully from 1,160 (57.7%) of the HBV-infected subjects. Finally, they found the interaction of rs1053004 with T1674C/G significantly increased the HCC risk^37^. Lately, the group performed a case-control study again with a larger sample size of 1,531 HCC patients and 2,489 HBV-positive subjects without HCC to evaluate the impacts of *HLA-DQ* SNPs (rs2856718 reported by a GWAS of chronic hepatitis B and rs9275319 reported by a HCC GWAS) and their interactions with HBV mutations on the risk of HCC. They sequenced the HBV EnhII/BCP/PC region successfully from 1,450 (36.11%) of all the HBV-infected subjects. And they found rs2856718 variant genotypes significantly decreased HCC risk and the variant genotypes of rs2856718 were significantly associated with an increased frequency of HBV A1726C mutation in genotype C. However, the association betweenrs9275319 and HCC risk was not observed (adjusted OR = 0.99, 95% CI = 0.83–1.18, *P* = 0.876), which failed to validate the results of the HCC GWAS (*P* = 2.72 × 10^−17^), and was different from our results (*P* = 1.008 × 10^−6^)^6^,^39^.The findings were exciting; however, the different study design (different sample size and matching criteria) and varying methodology (different models of nest multiplex PCR and primers, the detection rate and definition for hotspot mutations) could influence the reproducible of the results^13^,^15^,^18^,^19^,^33^,^37^,^39^,^40^.

To the best of our knowledge, this is the first large multi-centre study revealing that HBV genotype and mutations could affect HCC risk via interacting with *HLA-DQ/DR* genetic variants, and this is the first study constructing prediction models and estimating sensitivity and specificity. There are four advantages of our study. First, the multiple centres used included a large sample size matched by area, age and gender, and the rigid quality control provided sufficient statistical power and more convincing data. Second, because the HBV genotypes were varied among the controls, the controls from an ongoing large-scale, population-based cohort can be helpful for quality control. Third, the success rates of the determination of the HBV genotypes, subgenotypes and mutations were much higher than in previous studies, and the distribution of the HBV genotypes and subgenotypes between cases and controls was validated successfully in an additional independent sample set. Fourth, the diagnostic performance of the panel including the *HLA* SNPs, HBV genotype and mutations was relatively high (sensitivity = 81.3%, specificity = 74.8%), and the detection of these factors was non-invasive. However, this study had limitations as well. The sample size of the additional sample set was too small to validate the association of the HBV mutations or *HLA-DQ/DR* SNPs with HCC risk and the interaction of rs9272105 with the HBV genotype and mutations. Further studies with large sample size in diverse populations are warranted to validate and extend our findings. And HBV DNA levels, HBeAg status and ALT level were not detected in our case-control study because some participants had received antiviral treatments, especially for the HCC patients. In addition, since the controls were from a large-scale, population-based cohort, the sensitivity and specificity of the cirrhosis diagnosis may be relatively low, and the antiviral treatment scheme was not obtained. Therefore, cirrhosis status and antiviral therapy were also not shown. Further rigorous prospective studies were also needed to dynamically monitor the HBV persistent carriers. Since HBV EnhII/BCP/PC region is one of important regulatory regions for HBV replication and is less sensitive to antiviral treatments, we only amplified this region for HBV mutation analysis. The detection methods of HBV genotype and mutations were based on nested PCR and sequencing in this study, thus there might be a small number of HBV carriers who failed to detect HBV genotype and mutations for low viral loads. Moreover, we only focused on the HCC GWAS-identified *HLA* SNPs in terms of the host genetic polymorphism. Therefore, well-designed studies involving genome-wide genetic factors (at least immune-related genes), mutations of the entire HBV genome, and HBV DNA levels may improve the prediction accuracy.

## Methods

The methods were carried out in accordance with the approved guidelines. And all experimental protocols were approved by the institutional review board of Nanjing Medical University.

### Participants

The HCC patients were consecutively recruited between January 2006 and May 2014 at the First Affiliated Hospital of Nanjing Medical University (Nanjing, China), the Nantong Tumour Hospital (Nantong, China) and the Qidong Liver Cancer Institute (Qidong, China) from central and southern Jiangsu Province. The diagnosis of HCC was confirmed by a pathological examination and/or an alpha-fetoprotein elevation (>400 ng/ml) combined with an imaging examination (i.e., magnetic resonance imaging and/or computerised tomography). Because HCV infection is rare in Chinese populations, we excluded HCC patients with HCV infection. As a result, 1,507 HBV-related HCC cases consented to participate in the study and provided blood samples (535 cases from Nanjing, 522 cases from Nantong and 450 cases from Qidong). Only the 450 cases from Qidong were shared with the published GWAS^6^.

Because the cases were all from central and southern Jiangsu Province, the controls from three cities in central and southern Jiangsu Province were screened for being HBV persistent carriers in 2009 (48,417 subjects from Zhangjiagang, 43,563 subjects from Taixing and 57,192 subjects from Danyang) and were confirmed in 2010. All the participants were self-reported Han Chinese. The HBV persistent carriers were those subjects who were positive for both hepatitis B surface antigen (HBsAg) and antibodies against the hepatitis B core antigen (anti-HBc) but were negative for the HCV antibody (anti-HCV) at the two visits. Approximately 2,475 (5.11%), 3,413 (7.83%) and 5,587 (9.77%) HBV persistent carriers were identified from Zhangjiagang, Taixing and Danyang, respectively. Then, we selected 1,560 HBV persistent carriers from these three cities and matched them to the HCC cases by age and gender (510 controls from Zhangjiagang, 500 controls from Taixing and 550 controls from Danyang). These selected controls had no history of cancer by self-report and B ultrasonic scanning, and their demographic information, such as age and gender, was collected by face-to-face interviews. All the controls included in the current study were independent from the two published GWAS^5^,^6^.

An additional small sample set for validation of the HBV genotype main effect was consecutively recruited between December 2013 and April 2014 at the Tangdu and Xijing Hospital of the Fourth Military Medical University (Xi’an, China)in Shaanxi Province, including 107 HBV-related HCC cases and 204 HBV persistent carriers, in which age and gender were matched between the cases and controls.

After written informed consent was obtained, each participant was scheduled for an interview by using a structured questionnaire to collect demographic and exposure information. After interview, a venous blood sample of approximately 5 ml was collected from each participant.

### Serological testing

HBsAg, anti-HBs, anti-HBc, and anti-HCV were detected by an enzyme-linked immunosorbent assay (Kehua Bio-Engineering Co., Ltd., Shanghai, China) in the serum, following the manufacturer’s instructions as described previously^4^.

### Determination of HBV genotypes and subgenotypes

HBV DNA was extracted from 200 μl of serum of the HBV-positive samples using High Pure Viral Nucleic Acid kits (Roche Diagnostics GmbH, Mannheim, Germany) according to the manufacturer’s instructions. The HBV genotypes and subgenotypes were identified by nested multiplex PCR, which was modified from Cao *et al.*^13^.The first round of amplification with rTaq DNA polymerase (TaKaRa, Dalian, China) was carried out as followed: 1) P1-S and P1-AS primers were used for the amplification of the first round of the HBV genotypes A, B, C and D, 2) P2-S and P2-AS primers were used for the amplification of the HBV subgenotypes B1 and B2, and 3) P3-S and P3-AS primers were used for the amplification of the HBV genotypes E and F and the subgenotypes C1and C2. The product (2 μl) of the first round of PCR served as a template for the routine multiplex PCR. The primers used are listed in Supplementary Table S1. The PCR-amplified products were electrophoresed on a 2% agarose gel, stained with ethidium bromide and visualised under UV light. If the HBV genotype was determined successfully but the subgenotype was not, the genotype-specific product was sequenced and aligned with the reference sequences (http://www.ncbi.nlm.nih.gov; http://hbv.geno2pheno.org). All the genotyping assays were performed at the same time at the same lab without knowing the subjects’ case and control statuses; two blank (i.e., water) controls in each 96-well format were used for quality control, and more than 10% of the samples were randomly selected to be confirmed by DNA sequencing, yielding a 100% concordant. The success rates of determining the HBV genotypes and subgenotypes for each area were all above 96%.

### HBV mutation analysis

The HBV EnhII/BCP/PC region was amplified in the subjects with a successfully detected HBV genotype using nested PCR^13^. The primers for the first and second round of the nested PCR are listed in Supplementary Table S1. Direct DNA sequencing was carried out by using ABI PRISM BigDye sequencing kits and an ABI 3730 Genetic Analyser (Applied Biosystems, Foster City, CA). The HBV sequences were aligned and analysed using MEGA 5.0 software. After the alignment, the nucleotide with the highest frequency at each site in the HBV EnhII/BCP/PC region from the HBV persistent carriers was termed as the wild type nucleotide. Nucleotide substitutions with the other three nucleotides and deletions at each site were termed as mutations. A site with combined mutation frequencies >10% from all the participants was termed a hotspot.

### Genotyping of the *HLA-DP/DQ* SNPs

The genomic DNA was extracted from the leukocyte pellets by traditional proteinase K digestion, followed by phenol-chloroform extraction and ethanol precipitation. The SNPs rs9272105 and rs9275319 were genotyped by a TaqMan allelic discrimination assay on an ABI 7900 system (Applied Biosystems, La Jolla, CA). The information on the primers and probes is shown in Supplementary Table S1. All the genotyping assays were performed without knowing the subjects’ case and control statuses; two blank (i.e., water) controls in each 384-well format were used for quality control, and more than 10% of the samples were randomly selected to repeat, yielding a 100% concordant. The success rates of genotyping for the rs9272105 and rs9275319 SNPs were 98.73 and 98.79%, respectively.

### Statistical analysis

The differences of the demographic characteristics, HBV genotypes, subgenotypes and mutations frequencies, in addition to the genotype frequencies of the SNPs between the cases and controls, were calculated by Student’s *t-*tests or a one-way analysis of variance (for the continuous variables) and a χ^2^ test (for the categorical variables). The associations of the HBV genotype, mutations and the genotypes of the SNPs with HCC risk were estimated by computing odds ratios (ORs) and their 95% confidence intervals (CIs) from logistic regression analyses. The heterogeneity of the associations between subgroups was assessed by the χ^2^ -based Q test. The linkage disequilibrium (LD) information for the mutation hotspots was evaluated by a pairwise r^2^. A risk prediction model to classify the HCC cases and controls was constructed according to the following steps: (1) Prediction factor selection: HBV genotype, the 11 independent HCC-related mutations (C1653T, C1673T, T1674C/G, C1730G, A1752G, T1753C, A1762T, G1764A, G1899A, G1915A/C and C1969T), the *HLA* SNPs (rs9272105 and rs9275319), the HCC-related multiplicative interactions (rs9272105 with the HBV genotype, the HBV mutations C1673T, G1719T, A1726C, C1730G, A1752G and G1799C) and the main effect of the interactions were considered to be the predictive factors by conducting a backwards stepwise logistic regression model, with a significance level of 0.05 for removing the respective variables. (2) Risk model construction: the variables that remained in the backwards stepwise model were included, and the risk prediction model was constructed using a logistic regression model. (3) Risk model evaluation: the model performance was evaluated by receiver-operator characteristic (ROC) curves, and the area under the curve (AUC) was used to classify the HCC cases and controls. The sensitivity and specificity were calculated to illustrate the model effects using the “best threshold” criteria of the ROC curve. The difference in the area under two correlated ROC curves was evaluated by DeLong’s test. The model’s calibration was assessed by Hosmer-Lemeshow χ^2^ test. All the statistical analyses were performed with R software (version 2.13.0; The R Foundation for Statistical Computing), and *P* ≤ 0.05 in a two-sided test was considered statistically significant.

## Additional Information

**How to cite this article**: Wen, J. *et al.* Hepatitis B virus genotype, mutations, human leukocyte antigen polymorphisms and their interactions in hepatocellular carcinoma: a multi-centre case-control study. *Sci. Rep.* **5**, 16489; doi: 10.1038/srep16489 (2015).

## Supplementary Material

Supplementary Information

## Figures and Tables

**Table 1:** Distribution of HBV genotypes and subgenotypes in HCC and HBV persistent carriers.

Variable	HCC patients(n = 1507)N (%)	HBV persistentcarriers (n = 1560)N (%)	*P*
No. of HBV typed	1471 (97.6)	1536 (98.5)	0.089
Genotype			<0.001
C	1186 (80.6)	529 (34.4)	
B	159 (10.8)	629 (41.0)	
BC	121 (8.2)	375 (24.4)	
D	5 (0.3)	3 (0.2)	
Subgenotype			<0.001
C2	985 (67.0)	450 (29.3)	
C1C2	69 (4.7)	11 (0.7)	
C1	10 (0.7)	5 (0.3)	
C3	111 (7.5)	61 (4.0)	
C4	11 (0.7)	2 (0.1)	
B2	159 (10.8)	629 (41.0)	
B2C1	2 (0.1)	2 (0.1)	
B2C1C2	8 (0.5)	3 (0.2)	
B2C2	107 (7.3)	366 (23.8)	
B2C3	4 (0.3)	4 (0.3)	
D	5 (0.3)	3 (0.2)	

**Table 2:** Results of full model (including age, sex as adjustment) after stepwise regression analysis.

Variables	SE	Z	OR (95% CI)	*P*
Age	0.00	−1.34	0.99 (0.98–1.00)	0.180
Gender	0.11	−1.58	0.82 (0.63–1.05)	0.115
HBV genotype	0.24	9.52	2.48 (2.06–2.99)	<0.001
C1653T	0.28	5.33	2.06 (1.58–2.68)	<0.001
C1673T	0.44	2.48	1.82 (1.13–2.93)	0.013
T1674C/G	0.21	4.30	1.71 (1.34–2.18)	<0.001
C1730G	1.02	4.33	3.52 (1.99–6.23)	<0.001
A1752G	1.82	5.33	5.63 (2.98–10.62)	<0.001
T1753C	0.21	3.52	1.60 (1.23–2.08)	<0.001
A1762T	0.32	3.09	1.75 (1.23–2.49)	0.002
G1764A	0.48	4.67	2.47 (1.69–3.60)	<0.001
G1899A	0.21	3.32	1.57 (1.20–2.05)	0.001
G1915A/C	0.24	2.54	1.49 (1.10–2.04)	0.011
C1969T	0.31	4.00	1.92 (1.40–2.65)	<0.001
rs9275319	0.19	3.09	1.48 (1.15–1.89)	0.002
rs9272105	0.41	4.17	2.19 (1.52–3.17)	<0.001
A1752G * rs9272105	0.11	−3.21	0.52 (0.35–0.78)	0.001

Note: The references of the prediction factors were reset for consistent risk effect.

Abbreviation: SE, standard error; Z, Z value.
