# Supplementary material for: Hepatitis B virus genotype, mutations, human leukocyte antigen polymorphisms and their interactions in hepatocellular carcinoma: a multi-centre case-control study
Source: Sci Rep. 2015 Nov 16;5:16489. doi: 10.1038/srep16489 (PMC4644975; doi:10.1038/srep16489)
Supplement: Supplementary Information [file srep16489-s1.pdf]

Supplementary information

Hepatitis B virus genotype, mutations, human leukocyte antigen polymorphisms and their interactions in hepatocellular carcinoma: a multi-centre case-control study

Juan Wen, Ci Song, Deke Jiang, Tianbo Jin, Juncheng Dai, Liguo Zhu, Jiaze An, Yao Liu, Shijie Ma, Na Qin, Cheng Liang, Jiaping Chen, Yue Jiang, Linlin Yang, Jibin Liu, Li Liu, Tingting Geng, Chao Chen, ie Jiang, Jianguo Chen, Fengcai Zhu, Yefei Zhu, Long Yu, Hongbing Shen, Xiangjun Zhai, Jianfeng Xu, Zhibin Hu

Supplementary Figure

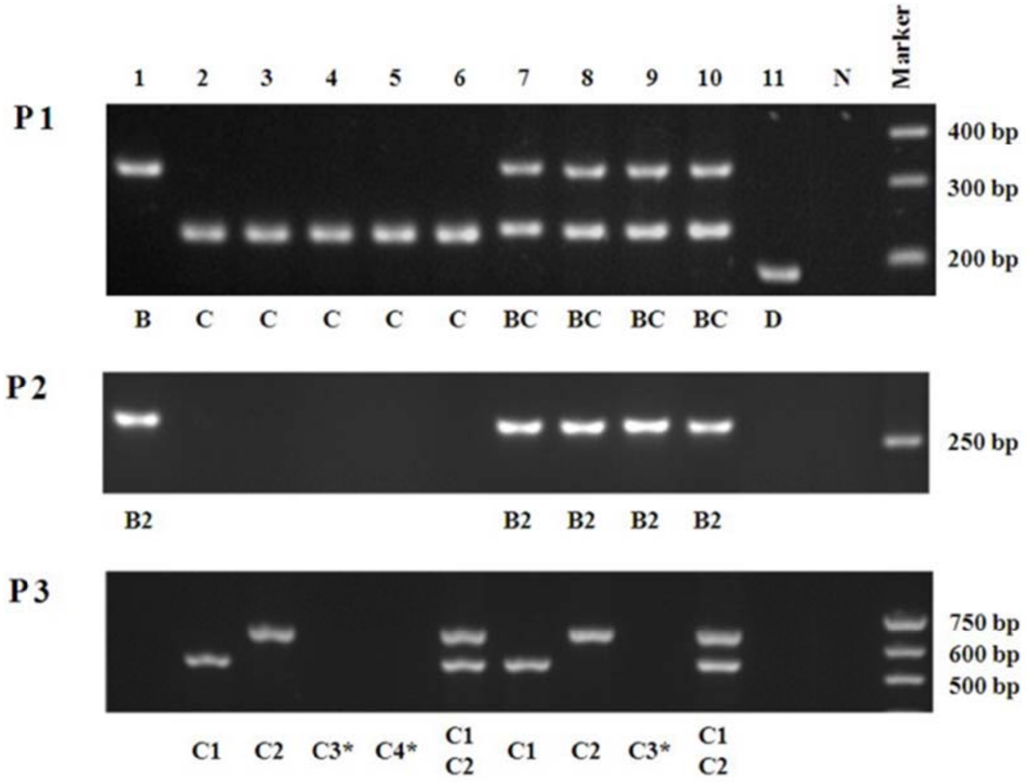

Supplementary Figure 1. The nested multiplex PCR specifically detects the HBV genotypes and subgenotypes from all the subjects, including the HCC patients and the HBV persistent carriers. Each lane corresponds to a subject. P1: Lane 1: genotype B; lanes 2-6: genotype C; lanes 7-10: genotype BC (coinfection); lane 11: genotype D. The sizes of the amplicons for the HBV genotypes are: genotype B: 331 bp; genotype C: 242 bp; genotype D: 189 bp. P2: Lanes 1, 7-10: subgenotype B2. P3: lanes 2, 7: subgenotype C1; lanes 3, 8: subgenotype C2; lanes 6, 10: subgenotype C1C2 (coinfection); lanes 4, 5, 9: subgenotypes C3, C4 and C3, respectively, which were detected by sequencing and alignment for the corresponding subgenotype of genotype C that was not detected. The sizes of the amplicons for the subgenotypes are: subgenotype B2: 278 bp; subgenotype C1: 554 bp; subgenotype C2: 652 bp.

A. HBV B type-specific sequence

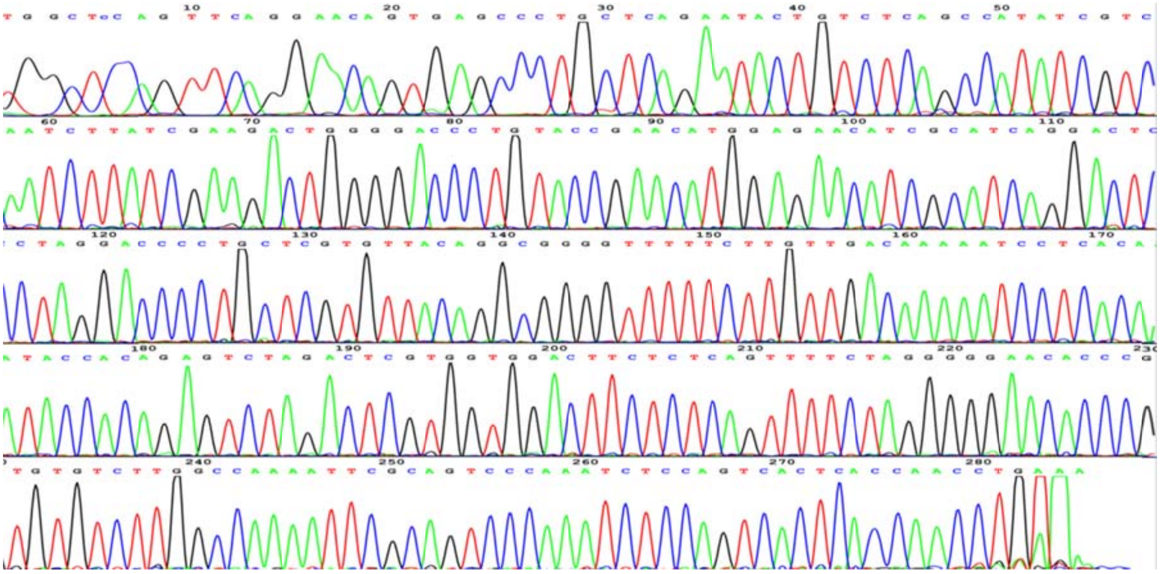

B. HBV C type-specific sequence

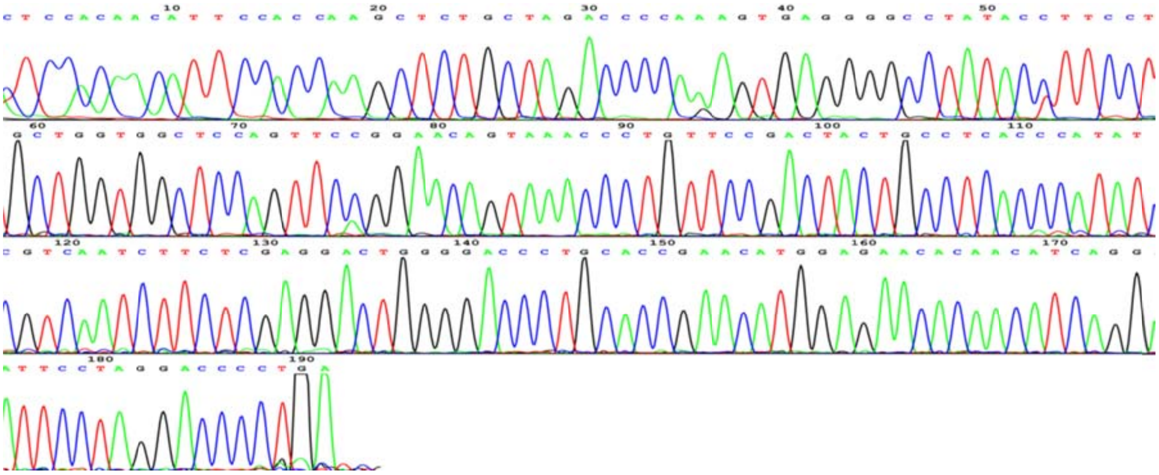

C. HBV D type-specific sequence

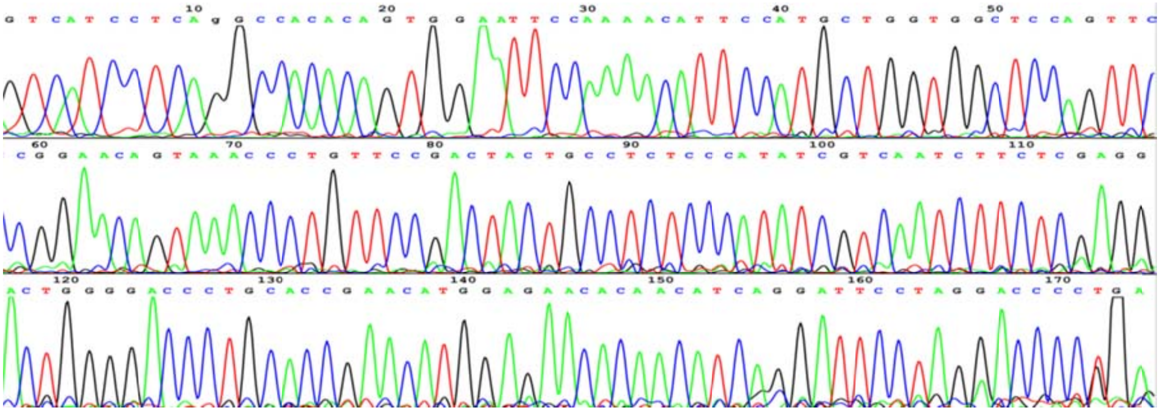

D. HBV B2 type-specific sequence

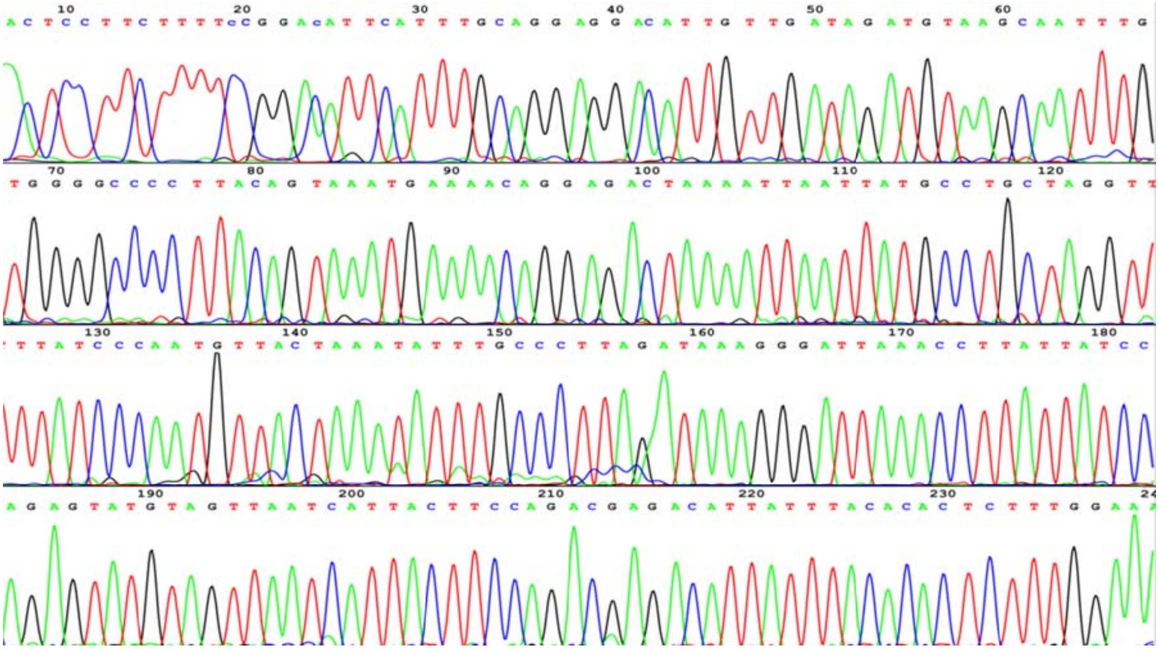

E. HBV C1 type-specific sequence

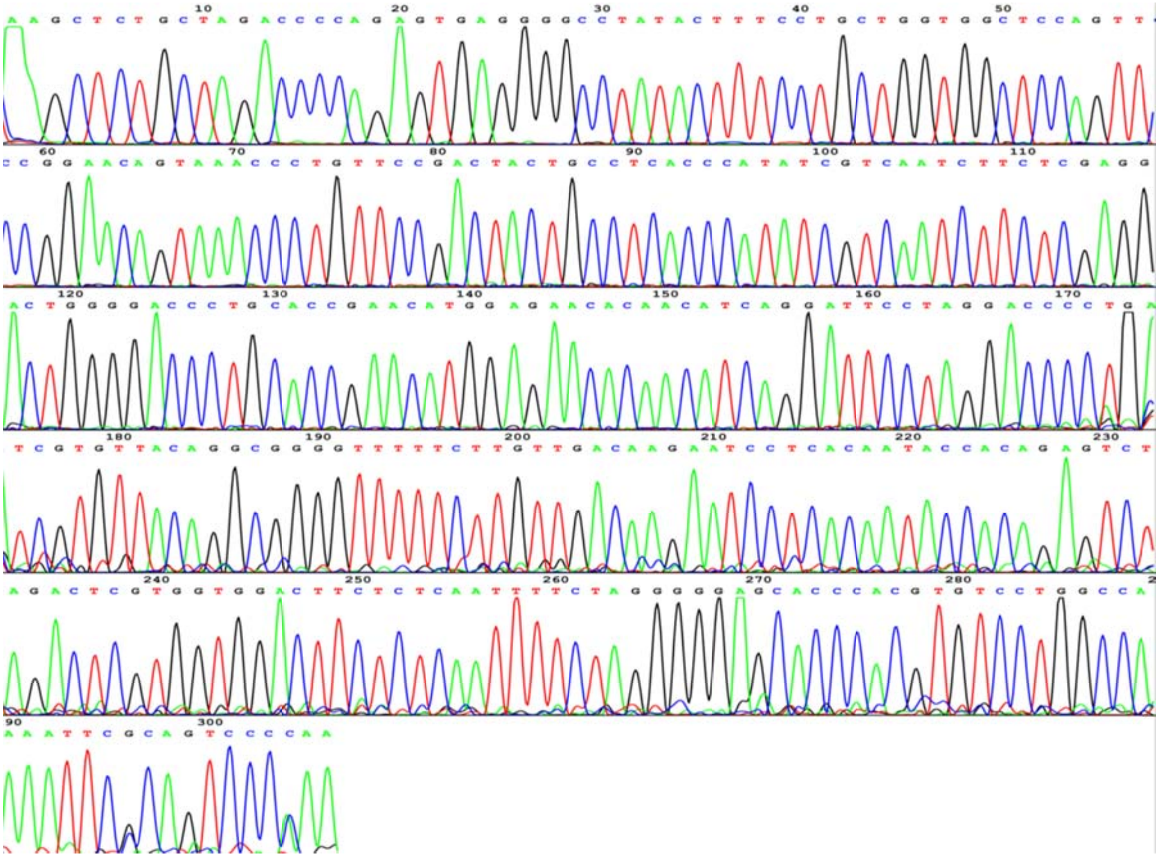

F. HBV C2 type-specific sequence

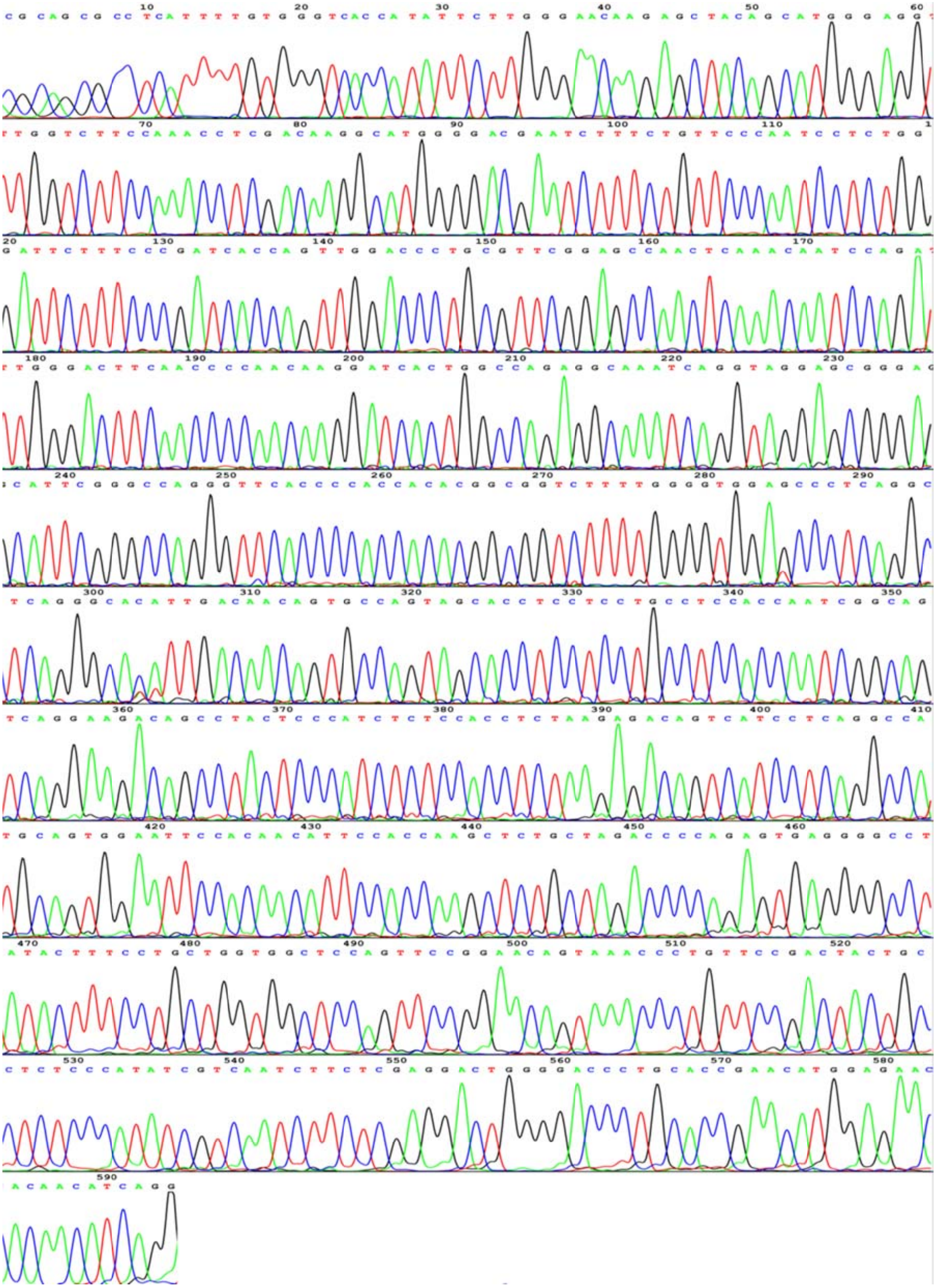

G. HBV C3 type-specific sequence

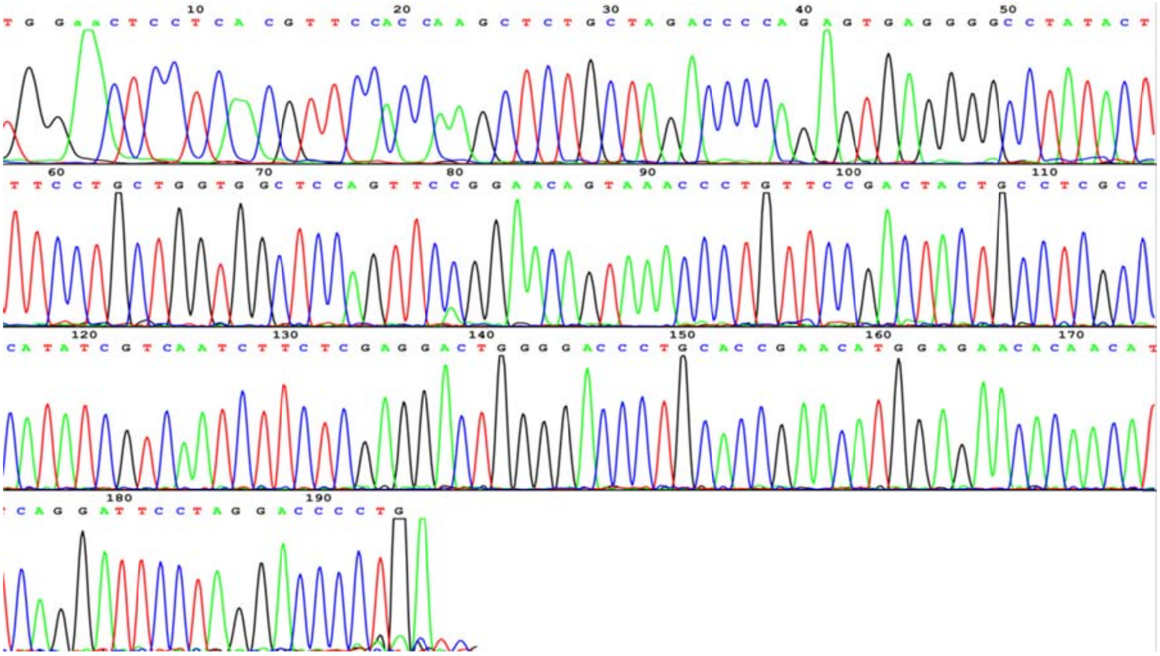

H. HBV C4 type-specific sequence

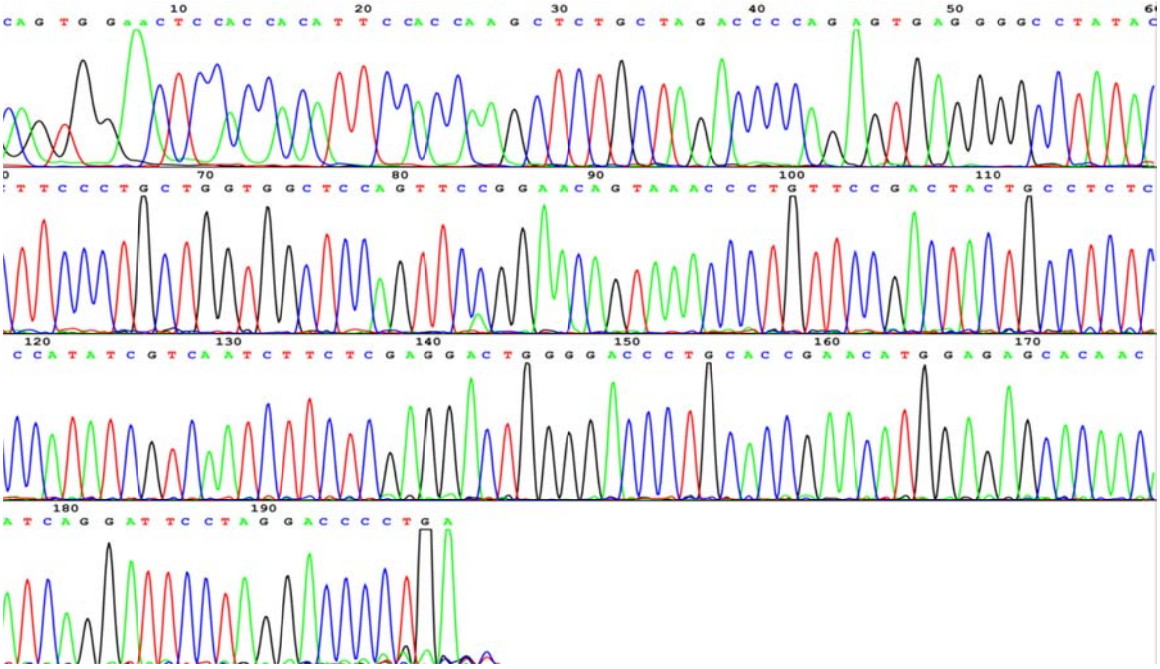

Supplementary Figure 2. HBV type-specific sequences including genotypes B and C and subgenotypes B2, C1, C2, C3 and C4. The sequences of subgenotypes C3 and C4 were obtained by sequencing the amplicon of genotype C.

A. C1653T

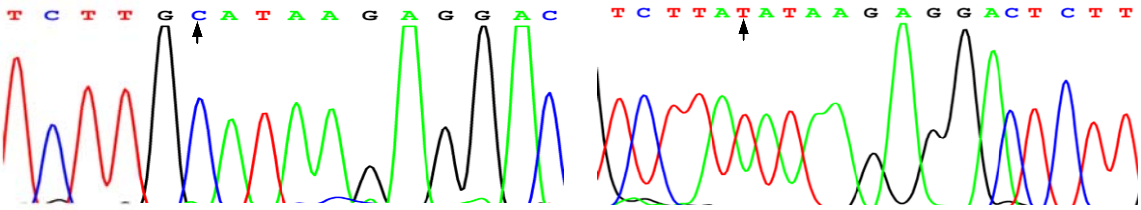

B. C1673T

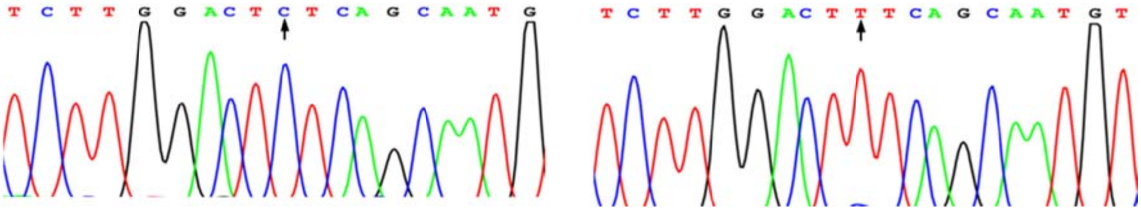

C. T1674C/G

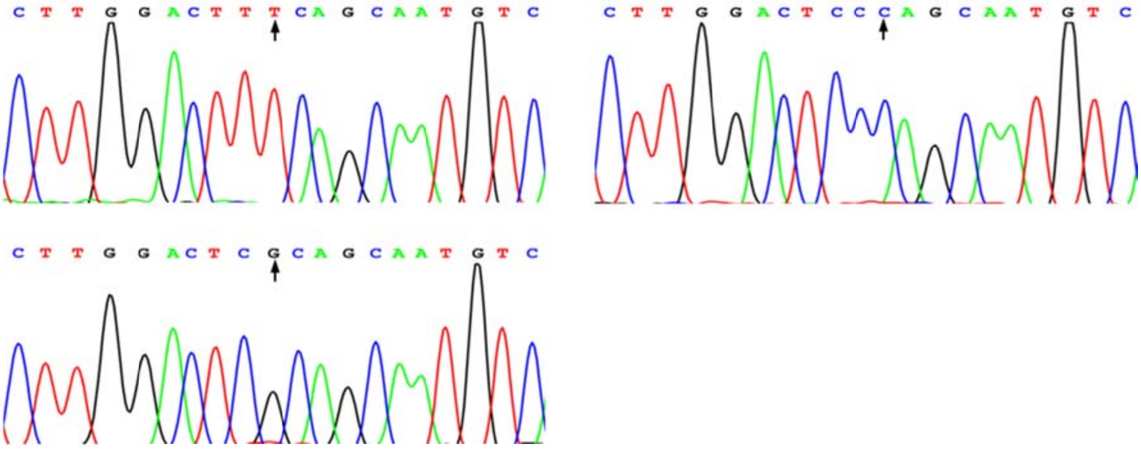

D. A1703G

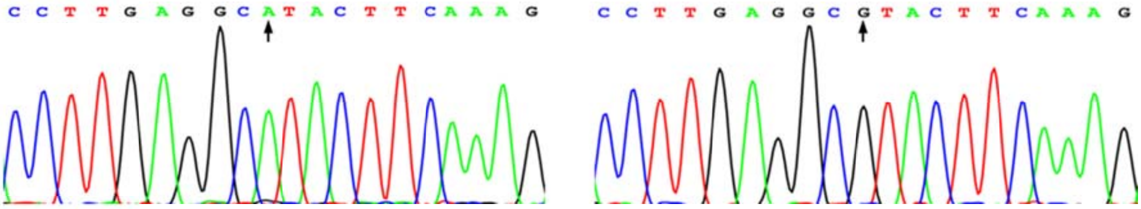

E. G1719T

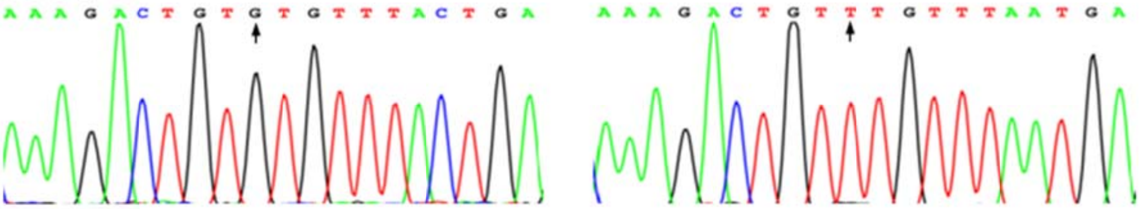

F. A1726C

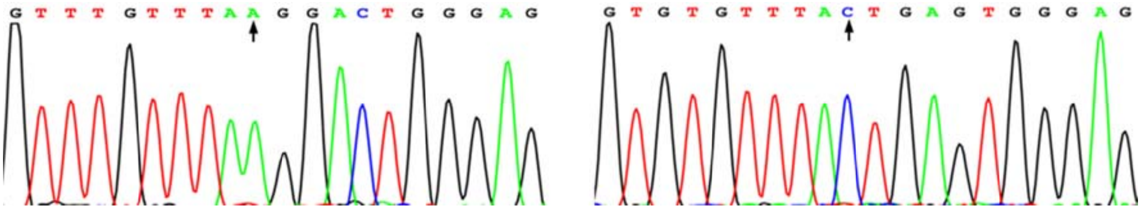

G. T1727A/G

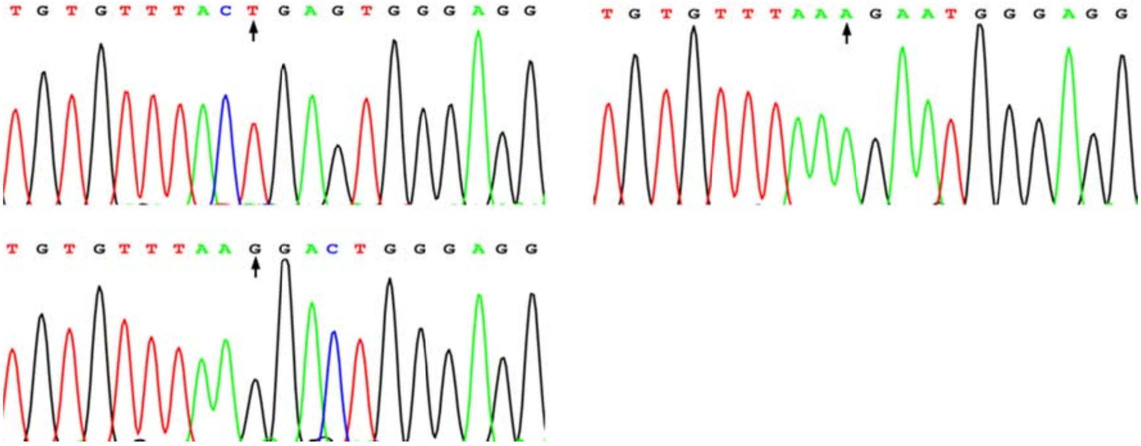

H. C1730G

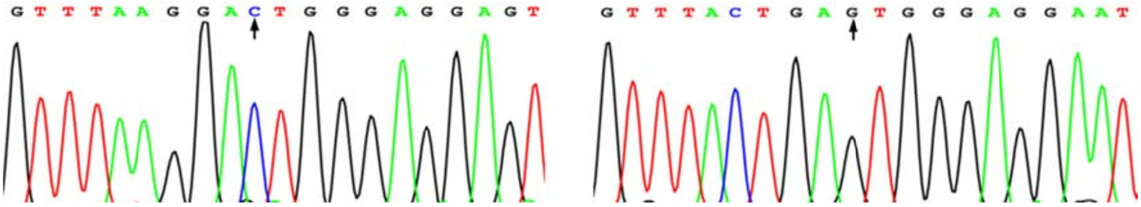

I. A1752G

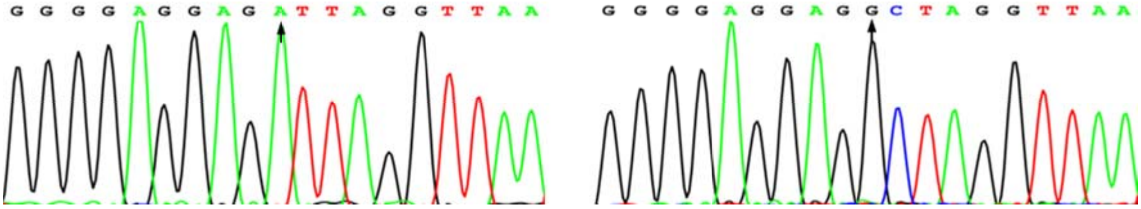

J. T1753C

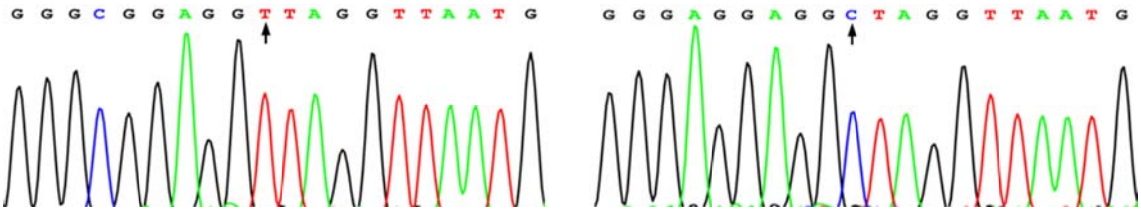

K. A1762T/G1764A

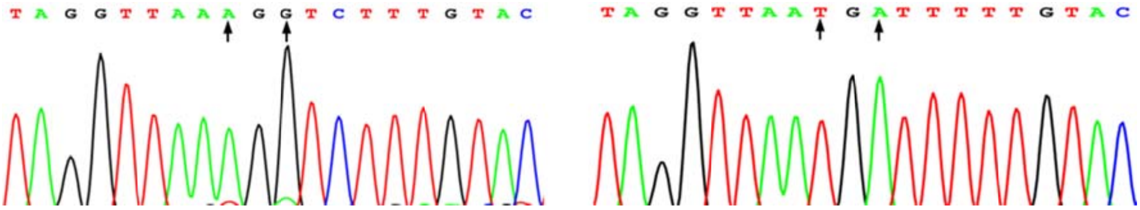

L. C1799G

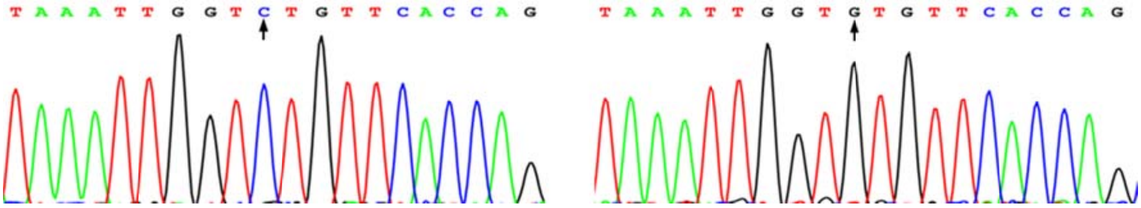

M. A1846T

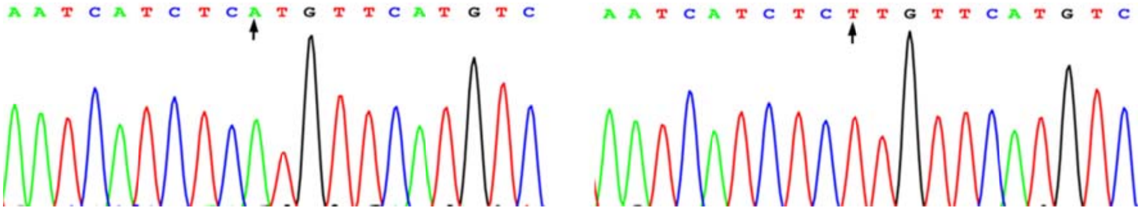

N. G1896A

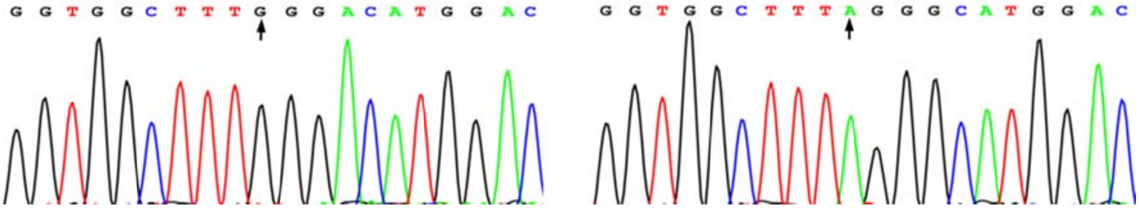

O. G1899A

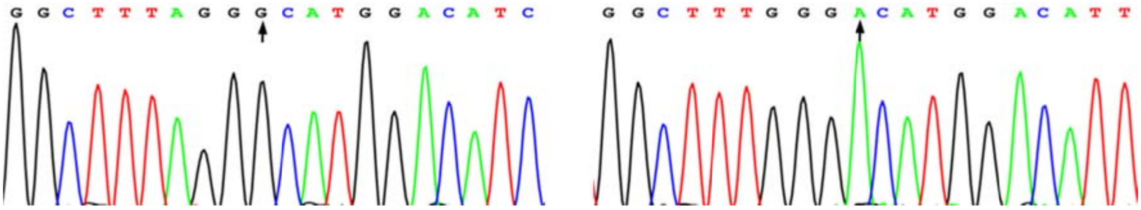

P. G1915A/C

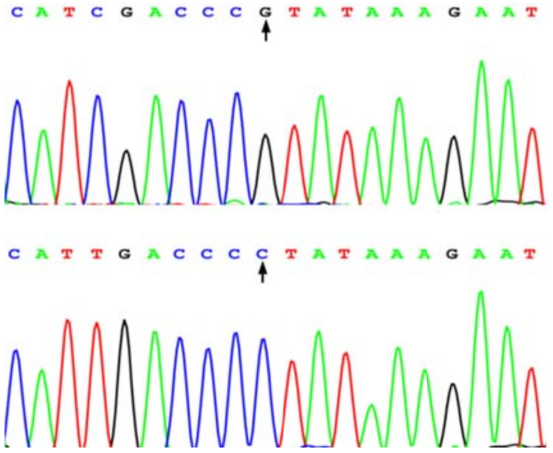

Q. C1969T

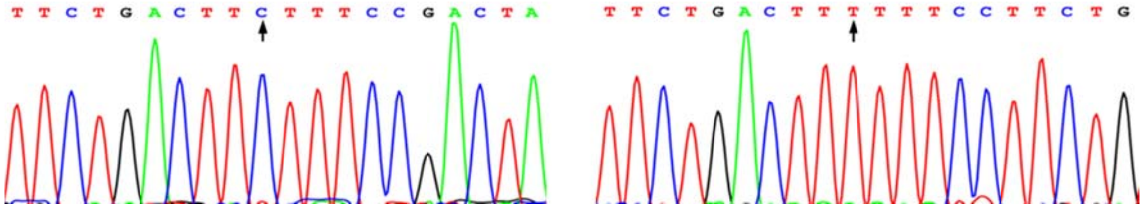

R. A1979G

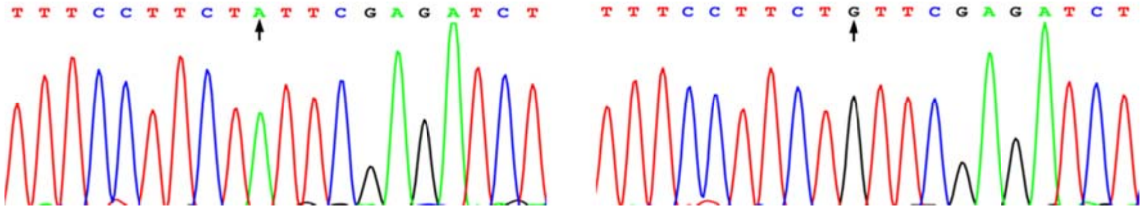

Supplementary Figure 3. The sequences of the HBV hotspot mutations in the EnhII/BCP/PC region including wild type and the mutation(s) of C1653T, C1673T, T1674C/G, A1703G, G1719T, A1726C, T1727A/G, C1730G, A1752G, T1753C, A1762T, G1764A, G1799C, A1846T, G1896A, G1899A, G1915A/C, C1969T and A1979G.

Supplementary table

Supplementary Table S1. Information of primers and probes

Type	Primers or probes (5'-3')	Position	Size (bp) of product
Nested PCR primers			
P1-S	TTTGCGGGTCACCATATTCTTGG	2815-2837	1105
P1-AS	CGAACCACTGAACAAATGGCACTAG	680-704	
P2-S	CCTACTGTTCAAGCCTCCAAGC	1856-1877	1026
P2-AS	GACCAACCTCCCATGCTGTAG	2861-2881	
P3-S	CCGGAAAGCTTGAGCTCTTCTTTTTCACCTCTGC CTAATCA	1821-1860	3259
P3-AS	CCGGAAAGCTTGAGCTCTTCAAAAAGTTGCATG GTGCTGG	1825-1864	
HBV Genotype primers			
A-S	GGCTCCAGTTCAGGAACAGT	64-83	68
A-AS	CTCGCGGAGATTGACGAGATGT	109-131	
B-S	CCAAACTCTTCAAGATCCCAGAGTCA	16-41	331
B-AS	ACAGGTTGGTGAGTGACTGGAGATTT	321-346	
C-S	CTCCCATCTCTCCACCTCTAAGAGACAGT	3164-3192	242
C-AS	CAGGGGTCCTAGGAATCCTGATGTTG	165-190	
D-S	CAGACGCCAACAAGGTAGGAGCT	2972-2994	189
D-AS	GAGTGTCTCTCAAAGGTGGAGACAGA	3135-3160	
E-S	ATACCCTATGGAAGGCGGGCATCT	2752-2775	130
E-AS	CCCATTCGAGAGGGACCGTCCA	2860-2881	
F-S	TATCTGTGGGTATCCATTTGAATACCTC	815-842	487
F-AS	CGAGCGAAACAGGCTGCAAG	1282-1301	
HBV Subgenotype primers			
B1-S	CCTTCTGACTTCTTTCCGTCGG	1958-1979	237
B1-AS	CCTGATCTTTAGGCCCATGTTAGTG	2170-2194	
B2-S	GGGCTTTATTCTTCTACGGTACCTTG	2487-2512	278
B2-AS	TTCCAAAGAGTGTGTAAATAATGTCTC	2738-2764	
C1-S	CATTGTTCACCTCACCATACAGCACTA	2040-2066	554
C1-AS	CATATTGTTGACATCTGTTGATAATATCCTCTTT	2560-2593	
C2-S	ATACTCTGTGGAAGGCTGGCATTC	2752-2775	652
C2-AS	GGGGTCCTAGGAATCCTGATGTTG	165-188	
Primers of enhancer II (EhnII), basic core promoter (BCP), precore regions			
BCP-1S	TGCACTTCGCTTCACCTCTG	1594-1613	736
BCP-1AS	TAAGCGGGAGGAGTGCGAAT	2310-2329	
BCP-2S	GCATGGAAACCACCGTGAAC	1606-1625	438
BCP-2AS	ATACAGAGCAGAGGCGGTAT	2024-2043	
Primers and probes for TaqMan allelic discrimination			
rs9272105-S	CAATTGTTTTTATAAGTTTCCTCTGCTTC		

rs9272105-AS	AGTCCTGTATGCTGATATCCAGTCAC
rs9272105-P-A	FAM-TGATTGTGAGAGACC-MGB
rs9272105-P-G	HEX-TGATTGTGAGAGGCC-MGB
rs9275319-S	GACAGTGCAGCCTTCAGTCTGT
rs9275319-AS	GCGGTCTTCCACCCTTCAT
rs9275319-P-C	FAM-TTGAAGGTCCCAGAGC-MGB
rs9275319-P-T	HEX-TGAAGGTCTCAGAGCC-MGB

Abbreviation: S, sense; AS, antisense.

Supplementary Table S2. Distribution of selected demographic variables in HCC and HBV persistent carriers

**Variable**	**HCC patients**			**HBV persistent carriers**			**All HCC patients (n = 1507) N (%)**	**All HBV persistent carriers (n = 1560) N (%)**	***P* [a]**
	Nanjing (n = 535) N (%)	Nantong (n = 522) N (%)	Qidong (n = 450) N (%)	Zhangjiagang (n = 510) N (%)	Taixing (n = 500) N (%)	Danyang (n = 550) N (%)			
Age	50.5±9.8	51.3±9.9	51.8±10.2	50.8±7.3	51.0±12.5	51.5±11.6	51.1±10.7	51.2±10.0	0.835
Gender									
Male	454 (84.9)	409 (78.4)	358 (79.6)	409 (80.2)	398 (79.6)	448 (81.5)	1221 (81.0)	1255 (80.4)	0.687
Female	81 (15.1)	113 (21.6)	92 (20.4)	101 (19.8)	102 (20.4)	102 (18.5)	286 (19.0)	305 (19.6)	

[a] All HCC patients vs. All HBV persistent carriers

Supplementary Table S3. Distribution of selected demographic variables in the additional independent sample set from Xi'an

Variable	HCC patients (n = 107) N (%)	HBV persistent carriers (n = 204) N (%)	*P*
Age	51.3±10.6	50.1±10.3	0.336
Gender			
Male	85 (79.4)	164 (80.4)	0.842
Female	22 (20.6)	40 (19.6)	

Supplementary Table S4. Distribution of HBV genotypes and subgenotypes in HCC and HBV persistent carriers from the central and southern Jiangsu Province

Variable	HCC patients			HBV persistent carriers		
	Nanjing (n = 535) N (%)	Nantong (n = 522) N (%)	Qidong (n = 450) N (%)	Zhangjiagang (n = 510) N (%)	Taixing (n = 500) N (%)	Danyang (n = 550) N (%)
No. of HBV typed	520 (97.2)	516 (98.9)	435 (96.7)	509 (99.8)	485 (97.0)	542 (98.5)
Genotype						
C	359 (69.0)	428 (82.9)	399 (91.7)	191 (37.5)	210 (43.3)	128 (23.6)
B	120 (23.1)	26 (5.0)	13 (3.0)	62 (12.2)	237 (48.9)	330 (60.9)
BC	40 (7.7)	61 (11.8)	20 (4.6)	255 (50.1)	36 (7.4)	84 (15.5)
D	1 (0.2)	1 (0.2)	3 (0.7)	1 (0.2)	2 (0.4)	0 (0.0)
Subgenotype						
C2	315 (60.6)	365 (70.7)	305 (70.1)	172 (33.8)	170 (35.1)	108 (19.9)
C1C2	8 (1.5)	27 (5.2)	34 (7.8)	6 (1.2)	1 (0.2)	4 (0.7)
C1	4 (0.8)	1 (0.2)	5 (1.1)	1 (0.2)	2 (0.4)	2 (0.4)
C3	28 (5.4)	28 (5.4)	55 (12.6)	10 (2.0)	37 (7.6)	14 (2.6)
C4	4 (0.8)	7 (1.4)	0 (0.0)	2 (0.4)	0 (0.0)	0 (0.0)
B2	120 (23.1)	26 (5.0)	13 (3.0)	62 (12.2)	237 (48.9)	330 (60.9)
B2C1	1 (0.2)	1 (0.2)	0 (0.0)	1 (0.2)	0 (0.0)	1 (0.2)
B2C1C2	0 (0.0)	4 (0.8)	4 (0.9)	2 (0.4)	1 (0.2)	0 (0.0)
B2C2	39 (7.5)	52 (10.1)	16 (3.7)	250 (49.1)	34 (7.0)	82 (15.1)
B2C3	0 (0.0)	4 (0.8)	0 (0.0)	2 (0.4)	1 (0.2)	1 (0.2)
D	1 (0.2)	1 (0.2)	3 (0.7)	1 (0.2)	2 (0.4)	0 (0.0)

Supplementary Table S5. Distribution of HBV genotypes and subgenotypes in the additional independent sample set from Xi'an

Variable	HCC patients (n = 107) N (%)	HBV persistent carriers (n = 204) N (%)	*P*
No. of HBV typed	103 (96.3)	197 (96.6)	0.889
Genotype			<0.001
C	80 (77.7)	109 (55.3)	
B	7 (6.8)	42 (21.3)	
BC	11 (10.7)	42 (21.3)	
D	5 (4.9)	4 (2.0)	
Subgenotype			0.004
C2	69 (67.0)	97 (49.2)	
C1C2	4 (3.9)	4 (2.0)	
C3	5 (4.9)	7 (3.6)	
C4	2 (1.9)	1 (0.5)	
B2	7 (6.8)	42 (21.3)	
B2C1	0 (0.0)	1 (0.5)	
B2C1C2	1 (1.0)	0 (0.0)	
B2C2	10 (9.7)	40 (20.3)	
B2C3	0 (0.0)	1 (0.5)	
D	5 (4.9)	4 (2.0)	

Supplementary Table S6. A nucleotide with the highest frequency at each site from HBV persistent carriers as wild type

Position	Wild type	Position	Wild type	Position	Wild type	Position	Wild type	Position	Wild type	Position	Wild type	Position	Wild type
**nt1653**	**C**	**nt1703**	**A**	**nt1753**	**T**	nt1803	T	nt1853	T	nt1903	G	nt1953	T
nt1654	A	nt1704	T	nt1754	T	nt1804	C	nt1854	G	nt1904	G	nt1954	T
nt1655	T	nt1705	A	nt1755	A	nt1805	A	nt1855	T	nt1905	A	nt1955	T
nt1656	A	nt1706	C	nt1756	G	nt1806	C	nt1856	C	nt1906	C	nt1956	T
nt1657	A	nt1707	T	nt1757	G	nt1807	C	nt1857	C	nt1907	A	nt1957	G
nt1658	G	nt1708	T	nt1758	T	nt1808	A	nt1858	T	nt1908	T	nt1958	C
nt1659	A	nt1709	C	nt1759	T	nt1809	G	nt1859	A	nt1909	T	nt1959	C
nt1660	G	nt1710	A	nt1760	A	nt1810	C	nt1860	C	nt1910	G	nt1960	T
nt1661	G	nt1711	A	nt1761	A	nt1811	A	nt1861	T	nt1911	A	nt1961	T
nt1662	A	nt1712	A	**nt1762**	**A**	nt1812	C	nt1862	G	nt1912	C	nt1962	C
nt1663	C	nt1713	G	nt1763	G	nt1813	C	nt1863	T	nt1913	C	nt1963	T
nt1664	T	nt1714	A	**nt1764**	**G**	nt1814	A	nt1864	T	nt1914	C	nt1964	G
nt1665	C	nt1715	C	nt1765	T	nt1815	T	nt1865	C	**nt1915**	**G**	nt1965	A
nt1666	T	nt1716	T	nt1766	C	nt1816	G	nt1866	A	nt1916	T	nt1966	C
nt1667	T	nt1717	G	nt1767	T	nt1817	C	nt1867	A	nt1917	A	nt1967	T
nt1668	G	nt1718	T	nt1768	T	nt1818	A	nt1868	G	nt1918	T	nt1968	T
nt1669	G	**nt1719**	**G**	nt1769	T	nt1819	A	nt1869	C	nt1919	A	**nt1969**	**C**
nt1670	A	nt1720	T	nt1770	G	nt1820	C	nt1870	C	nt1920	A	nt1970	T
nt1671	C	nt1721	G	nt1771	T	nt1821	T	nt1871	T	nt1921	A	nt1971	T
nt1672	T	nt1722	T	nt1772	A	nt1822	T	nt1872	C	nt1922	G	nt1972	T
**nt1673**	**C**	nt1723	T	nt1773	C	nt1823	T	nt1873	C	nt1923	A	nt1973	C
**nt1674**	**T**	nt1724	T	nt1774	T	nt1824	T	nt1874	A	nt1924	A	nt1974	C
nt1675	C	nt1725	A	nt1775	A	nt1825	T	nt1875	A	nt1925	T	nt1975	T
nt1676	A	**nt1726**	**A**	nt1776	G	nt1826	C	nt1876	G	nt1926	T	nt1976	T
nt1677	G	**nt1727**	**T**	nt1777	G	nt1827	A	nt1877	C	nt1927	T	nt1977	C
nt1678	C	nt1728	G	nt1778	A	nt1828	C	nt1878	T	nt1928	G	nt1978	T
nt1679	A	nt1729	A	nt1779	G	nt1829	C	nt1879	G	nt1929	G	**nt1979**	**A**
nt1680	A	**nt1730**	**C**	nt1780	G	nt1830	T	nt1880	T	nt1930	A	nt1980	T
nt1681	T	nt1731	T	nt1781	C	nt1831	C	nt1881	G	nt1931	G	nt1981	T
nt1682	G	nt1732	G	nt1782	T	nt1832	T	nt1882	C	nt1932	C	nt1982	C
nt1683	T	nt1733	G	nt1783	G	nt1833	G	nt1883	C	nt1933	T	nt1983	G
nt1684	C	nt1734	G	nt1784	T	nt1834	C	nt1884	T	nt1934	T	nt1984	A
nt1685	A	nt1735	A	nt1785	A	nt1835	C	nt1885	T	nt1935	C	nt1985	G
nt1686	A	nt1736	G	nt1786	G	nt1836	T	nt1886	G	nt1936	T	nt1986	A
nt1687	C	nt1737	G	nt1787	G	nt1837	A	nt1887	G	nt1937	G	nt1987	T
nt1688	G	nt1738	A	nt1788	C	nt1838	A	nt1888	G	nt1938	T	nt1988	C
nt1689	A	nt1739	G	nt1789	A	nt1839	T	nt1889	T	nt1939	G	nt1989	T
nt1690	C	nt1740	T	nt1790	T	nt1840	C	nt1890	G	nt1940	G	nt1990	C
nt1691	C	nt1741	T	nt1791	A	nt1841	A	nt1891	G	nt1941	A	nt1991	C
nt1692	G	nt1742	G	nt1792	A	nt1842	T	nt1892	C	nt1942	G	nt1992	T
nt1693	A	nt1743	G	nt1793	A	nt1843	C	nt1893	T	nt1943	T	nt1993	C
nt1694	C	nt1744	G	nt1794	T	nt1844	T	nt1894	T	nt1944	T	nt1994	G
nt1695	C	nt1745	G	nt1795	T	nt1845	C	nt1895	T	nt1945	A	nt1995	A
nt1696	T	nt1746	G	nt1796	G	**nt1846**	**A**	**nt1896**	**G**	nt1946	C	nt1996	T
nt1697	T	nt1747	A	nt1797	G	nt1847	T	nt1897	G	nt1947	T	nt1997	A
nt1698	G	nt1748	G	nt1798	T	nt1848	G	nt1898	G	nt1948	C	nt1998	C
nt1699	A	nt1749	G	**nt1799**	**G**	nt1849	T	**nt1899**	**G**	nt1949	T	nt1999	C
nt1700	G	nt1750	A	nt1800	T	nt1850	T	nt1900	C	nt1950	C	nt2000	G
nt1701	G	nt1751	G	nt1801	G	nt1851	C	nt1901	A	nt1951	T		
nt1702	C	**nt1752**	**A**	nt1802	T	nt1852	A	nt1902	T	nt1952	T		

Note: The bold represents hotspot sites with combined mutation frequencies > 10% from all the participants.

Supplementary Table S7. The associations of the hotspot mutations in the EnhII/BCP/PC region of HBV in HCC patients, as compared with HBV persistent carriers

Substitutions	HCC patients (n = 1471) N (%)	HBV persistent carriers (n = 1536) N (%)	OR (95%CI) [a]	*P* [a]	OR (95%CI) [b]	*P* [b]
C1653T						
C	928 (70.7)	1273 (90.1)	1.00		1.00	
T	385 (29.3)	140 (9.9)	3.78 (3.06-4.67)	5.82×10^{-35}	2.17 (1.67-2.80)	4.48×10^{-9}
C1673T						
C	1151 (87.9)	766 (55.1)	1.00		1.00	
T	158 (12.1)	624 (44.9)	0.17 (0.14-0.20)	1.97×10^{-70}	0.57 (0.35-0.92)	2.05×10^{-2}
T1674C/G						
T	857 (65.6)	1244 (88.2)	1.00		1.00	
C/G	449 (34.4)	167 (11.8)	3.91 (3.21-4.76)	1.55×10^{-41}	1.90 (1.49-2.42)	2.79×10^{-7}
A1703G						
A	1155 (88.3)	1291 (91.6)	1.00		1.00	
G	153 (11.7)	119 (8.4)	1.44 (1.12-1.85)	4.96×10^{-3}	0.94 (0.68-1.29)	7.04×10^{-1}
G1719T						
G	324 (24.7)	816 (57.7)	1.00		1.00	
T	989 (75.3)	597 (42.3)	4.19 (3.56-4.94)	1.48×10^{-65}	1.12 (0.81-1.55)	4.96×10^{-1}
A1726C						
A	1174 (89.4)	866 (61.3)	1.00		1.00	
C	139 (10.6)	547 (38.7)	0.19 (0.15-0.23)	2.59×10^{-57}	0.82 (0.51-1.32)	4.17×10^{-1}
T1727A/G						
T	169 (12.9)	671 (47.6)	1.00		1.00	
A/G	1144 (87.1)	740 (52.4)	6.17 (5.09-7.48)	1.68×10^{-76}	1.19 (0.67-2.12)	5.50×10^{-1}
C1730G						
C	1140 (87.5)	778 (55.6)	1.00		1.00	
G	163 (12.5)	622 (44.4)	0.18 (0.15-0.22)	3.86×10^{-67}	2.07 (1.02-4.20)	4.47×10^{-2}
A1752G						
A	1211 (92.6)	925 (65.7)	1.00		1.00	
G	97 (7.4)	482 (34.3)	0.15 (0.12-0.19)	1.82×10^{-55}	0.31 (0.20-0.48)	7.63×10^{-8}
T1753C						
T	896 (70.3)	1213 (88.7)	1.00		1.00	
C	378 (29.7)	154 (11.3)	3.33 (2.71-4.10)	3.24×10^{-30}	1.67 (1.29-2.15)	8.01×10^{-5}
A1762T						
A	228 (17.4)	761 (53.9)	1.00		1.00	
T	1085 (82.6)	652 (46.1)	5.56 (4.66-6.64)	2.07×10^{-80}	1.85 (1.31-2.61)	4.72×10^{-4}
G1764A						
G	160 (12.2)	723 (51.2)	1.00		1.00	
A	1151 (87.8)	690 (48.8)	7.57 (6.23-9.21)	2.58×10^{-91}	2.60 (1.79-3.77)	4.45×10^{-7}
G1799C						
G	171 (13.0)	651 (46.1)	1.00		1.00	
C	1142 (87.0)	761 (53.9)	5.74 (4.74-6.96)	3.66×10^{-71}	1.15 (0.65-2.06)	6.33×10^{-1}
A1846T						
A	881 (67.8)	991 (70.9)	1.00		1.00	
T	419 (32.2)	406 (29.1)	1.16 (0.99-1.37)	7.48×10^{-2}	0.80 (0.63-1.02)	7.46×10^{-2}
G1896A						
G	635 (48.4)	731 (51.7)	1.00		1.00	
A	677 (51.6)	682 (48.3)	1.14 (0.98-1.33)	8.04×10^{-2}	1.18 (0.96-1.46)	1.19×10^{-1}

G1899A						
G	1001 (76.2)	1221 (86.4)	1.00		1.00	
A	312 (23.8)	192 (13.6)	1.99 (1.63-2.42)	1.32×10^{-11}	1.77 (1.35-2.32)	3.33×10^{-5}
G1915A/C						
G	1123 (85.8)	1267 (90.2)	1.00		1.00	
A/C	186 (14.2)	137 (9.8)	1.53 (1.21-1.94)	3.62×10^{-4}	1.48 (1.08-2.03)	1.40×10^{-2}
C1969T						
C	1102 (83.9)	1328 (94.0)	1.00		1.00	
T	211 (16.1)	85 (6.0)	2.99 (2.30-3.89)	4.39×10^{-16}	2.01 (1.47-2.76)	1.36×10^{-5}
A1979G						
A	1126 (85.8)	1237 (87.7)	1.00		1.00	
G	187 (14.2)	174 (12.3)	1.18 (0.95-1.47)	1.44×10^{-1}	1.06 (0.79-1.41)	7.06×10^{-1}

[a] Logistic regression analyses adjusted for age and gender between HCC patients and HBV persistent carriers.

[b] Logistic regression analyses adjusted for age, gender and all the other mutations between HCC patients and HBV persistent carriers.

Supplementary Table S8. The LD information (pairwise r^2) for the hotspot mutations in the EnhII/BCP/PC region of HBV

Mutations	C1653T	C1673T	T1674C/G	A1703G	G1719T	A1726C	T1727A/G	C1730G	A1752G	T1753C	A1762T	G1764A	G1799C	A1846T	G1896A	G1899A	G1915A/C	C1969T
C1653T	-	-	-	-	-	-	-	-	-	-	-	-	-	-	-	-	-	-
C1673T	0.042	-	-	-	-	-	-	-	-	-	-	-	-	-	-	-	-	-
T1674C/G	0.013	0.097	-	-	-	-	-	-	-	-	-	-	-	-	-	-	-	-
A1703G	0.000	0.029	0.008	-	-	-	-	-	-	-	-	-	-	-	-	-	-	-
G1719T	0.038	0.514	0.149	0.055	-	-	-	-	-	-	-	-	-	-	-	-	-	-
A1726C	0.036	0.596	0.081	0.024	0.436	-	-	-	-	-	-	-	-	-	-	-	-	-
T1727A/G	0.049	0.714	0.107	0.032	0.554	0.704	-	-	-	-	-	-	-	-	-	-	-	-
C1730G	0.046	0.762	0.097	0.031	0.550	0.701	0.822	-	-	-	-	-	-	-	-	-	-	-
A1752G	0.032	0.519	0.074	0.021	0.364	0.402	0.551	0.604	-	-	-	-	-	-	-	-	-	-
T1753C	0.014	0.049	0.014	0.011	0.042	0.038	0.053	0.048	0.030	-	-	-	-	-	-	-	-	-
A1762T	0.044	0.064	0.026	0.004	0.072	0.053	0.082	0.059	0.016	0.084	-	-	-	-	-	-	-	-
G1764A	0.052	0.096	0.054	0.003	0.118	0.081	0.122	0.093	0.031	0.078	0.735	-	-	-	-	-	-	-
G1799C	0.043	0.717	0.106	0.031	0.547	0.633	0.797	0.846	0.585	0.044	0.064	0.095	-	-	-	-	-	-
A1846T	0.013	0.013	0.001	0.000	0.013	0.024	0.017	0.027	0.005	0.001	0.007	0.008	0.025	-	-	-	-	-
G1896A	0.008	0.010	0.000	0.004	0.008	0.025	0.017	0.029	0.006	0.001	0.002	0.005	0.024	0.135	-	-	-	-
G1899A	0.014	0.001	0.001	0.001	0.001	0.003	0.001	0.002	0.000	0.013	0.013	0.017	0.004	0.112	0.005	-	-	-
G1915A/C	0.001	0.014	0.003	0.001	0.019	0.012	0.017	0.015	0.010	0.002	0.000	0.000	0.014	0.006	0.000	0.004	-	-
C1969T	0.006	0.015	0.008	0.014	0.020	0.016	0.021	0.015	0.009	0.006	0.008	0.013	0.015	0.001	0.003	0.007	0.000	-
A1979G	0.000	0.000	0.000	0.000	0.002	0.001	0.001	0.002	0.000	0.005	0.004	0.005	0.002	0.004	0.033	0.000	0.000	0.003

Supplementary Table S9. Association between two SNPs and HBV-related HCC susceptibility

Genotype	HCC patients			HBV persistent carriers			All HCC patients (n = 1507) N (%)	All HBV persistent carriers (n = 1560) N (%)	OR (95%CI) [a]	*P* [a]
	Nanjing (n = 535) N (%)	Nantong (n = 522) N (%)	Qidong (n = 450) N (%)	Zhangjiagang (n = 510) N (%)	Taixing (n = 500) N (%)	Danyang (n = 550) N (%)				
rs9272105										
G/G	131 (24.9)	133 (25.8)	111 (24.7)	152 (30.0)	158 (32.2)	180 (33.3)	375 (25.2)	490 (31.9)	1.00	
G/A	237 (45.1)	266 (51.7)	216 (48.1)	246 (48.6)	244 (49.7)	258 (47.7)	719 (48.3)	748 (48.6)	1.25 (1.06-1.49)	8.793×10^{-3}
A/A	158 (30.0)	116 (22.5)	122 (27.2)	108 (21.3)	89 (18.1)	103 (19.0)	396 (26.6)	300 (19.5)	1.72 (1.41-2.11)	1.158×10^{-7}
Dominant									1.39 (1.19-1.63)	4.703×10^{-5}
Recessive									1.50 (1.26-1.77)	3.472×10^{-6}
Additive									1.31 (1.18-1.45)	1.183×10^{-7}
rs9275319										
A/A	456 (87.0)	434 (83.6)	374 (83.1)	383 (75.4)	377 (76.9)	438 (81.3)	1264 (84.7)	1198 (77.9)	1.00	
G/A	65 (12.4)	81 (15.6)	71 (15.8)	117 (23.0)	105 (21.4)	91 (16.9)	217 (14.5)	313 (20.4)	0.66 (0.54-0.79)	1.491×10^{-5}
G/G	3 (0.6)	4 (0.8)	5 (1.1)	8 (1.6)	8 (1.6)	10 (1.9)	12 (0.8)	26 (1.7)	0.44 (0.22-0.88)	1.928×10^{-2}
Dominant									0.64 (0.53-0.77)	1.969×10^{-6}
Recessive									0.47 (0.24-0.94)	2.718×10^{-2}
Additive									0.66 (0.56-0.78)	1.008×10^{-6}

[a] Logistic regression analyses adjusted for age and gender between all HCC patients and all HBV persistent carriers.

Supplementary Table S10. Stratified analyses on rs9272105 and rs9275319 with HCC susceptibility

HBV genotype	HCC patients	HBV persistent carriers	OR (95% CI)	*P* [a]
rs9272105 (GG / GA / AA)				
Stratification model 1				
B	32 /67 /55	182 /313 /122	1.63 (1.27-2.10)	0.118
BC	20 /69 /31	126 /174 /72	1.62 (1.20-2.18)	
C	312 /566 /297	170 /252 /101	1.26 (1.09-1.46)	
Stratification model 2				
B-related [b]	52 /136 /86	308 /487 /194	1.61 (1.33-1.95)	0.046
Non-B [c]	171 /253 /102	312 /569 /299	1.26 (1.09-1.46)	
rs9275319 (AA / GA / GG)				
Stratification model 1				
B	138 /18 / 1	496 /110 /10	0.58 (0.36-0.94)	0.949
BC	101 /19 / 1	282 /80 /9	0.64 (0.39-1.04)	
C	989 /176 /9	399 /118 /6	0.63 (0.50-0.80)	
Stratification model 2				
B-related [b]	239 /37 /2	778 /190 /19	0.62 (0.44-0.88)	0.883
Non-B [c]	994 /176 /9	402 /118 /6	0.64 (0.50-0.81)	

Note: Logistic regression analyses adjusted for age and gender in the additive genetic model.

[a] P-value for the heterogeneity test.

[b] B-related genotypes including B, BC.

[c] Non-B genotypes including C, D.

Supplementary Table S11. Contributions of the multiplicative interactions of HLA SNPs with HBV mutations in the EnhII/BCP/PC region of HBV to HCC risk

SNP * Mutation	OR (95%CI)	*P*	SNP * Mutation	OR (95%CI)	*P*
rs9272105 * C1653T	0.88 (0.66-1.19)	0.417	rs9275319 * C1653T	1.43 (0.84-2.45)	0.189
rs9272105 * C1673T	1.43 (1.07-1.90)	0.016	rs9275319 * C1673T	0.76 (0.45-1.31)	0.327
rs9272105 * T1674C/G	0.90 (0.68-1.18)	0.442	rs9275319 * T1674C/G	0.77 (0.49-1.21)	0.260
rs9272105 * A1703G	1.00 (0.69-1.43)	0.979	rs9275319 * A1703G	0.94 (0.52-1.71)	0.844
rs9272105 * G1719T	0.76 (0.60-0.96)	0.020	rs9275319 * G1719T	1.07 (0.71-1.61)	0.747
rs9272105 * A1726C	1.38 (1.02-1.86)	0.039	rs9275319 * A1726C	0.78 (0.45-1.35)	0.374
rs9272105 * T1727A/G	0.75 (0.57-1.00)	0.051	rs9275319 * T1727A/G	1.26 (0.75-2.10)	0.389
rs9272105 * C1730G	1.36 (1.02-1.81)	0.035	rs9275319 * C1730G	0.90 (0.53-1.52)	0.692
rs9272105 * A1752G	1.84 (1.30-2.61)	0.001	rs9275319 * A1752G	0.57 (0.28-1.18)	0.131
rs9272105 * T1753C	1.06 (0.78-1.43)	0.726	rs9275319 * T1753C	1.02 (0.64-1.63)	0.937
rs9272105 * A1762T	0.96 (0.75-1.24)	0.763	rs9275319 * A1762T	1.36 (0.86-2.16)	0.191
rs9272105 * G1764A	0.77 (0.58-1.01)	0.063	rs9275319 * G1764A	1.09 (0.66-1.81)	0.725
rs9272105 * G1799C	0.70 (0.53-0.93)	0.014	rs9275319 * G1799C	1.16 (0.69-1.95)	0.574
rs9272105 * A1846T	1.17 (0.92-1.48)	0.191	rs9275319 * A1846T	0.77 (0.51-1.15)	0.205
rs9272105 * G1896A	0.94 (0.76-1.17)	0.590	rs9275319 * G1896A	0.83 (0.58-1.20)	0.321
rs9272105 * G1899A	1.11 (0.84-1.48)	0.450	rs9275319 * G1899A	1.02 (0.62-1.68)	0.935
rs9272105 * G1915A/C	1.15 (0.82-1.60)	0.415	rs9275319 * G1915A/C	1.24 (0.72-2.14)	0.431
rs9272105 * C1969T	1.26 (0.85-1.86)	0.243	rs9275319 * C1969T	1.22 (0.64-2.32)	0.551
rs9272105 * A1979G	1.01 (0.74-1.40)	0.928	rs9275319 * A1979G	1.45 (0.84-2.52)	0.187

Note: Logistic regression analyses adjusted for age and gender in the additive genetic model.
